# Enabled Negatively Regulates Diaphanous-Driven Actin Dynamics In Vitro and In Vivo

**DOI:** 10.1016/j.devcel.2014.01.015

**Published:** 2014-02-24

**Authors:** Colleen G. Bilancia, Jonathan D. Winkelman, Denis Tsygankov, Stephanie H. Nowotarski, Jennifer A. Sees, Kate Comber, Iwan Evans, Vinal Lakhani, Will Wood, Timothy C. Elston, David R. Kovar, Mark Peifer

**Affiliations:** 1 Biology Department, University of North Carolina at Chapel Hill, Chapel Hill, NC 27599, USA; 2 Lineberger Comprehensive Cancer Center, University of North Carolina at Chapel Hill, Chapel Hill, NC 27599, USA; 3 Department of Molecular Genetics and Cell Biology, The University of Chicago, Chicago, IL 60637, USA; 4 Department of Pharmacology, University of North Carolina at Chapel Hill, Chapel Hill, NC 27599, USA; 5 Department of Biology and Biochemistry, University of Bath, Bath BA2 7AY, UK; 6 Department of Biochemistry and Molecular Biology, The University of Chicago, Chicago, IL 60637, USA

## Abstract

Actin regulators facilitate cell migration by controlling cell protrusion architecture and dynamics. As the behavior of individual actin regulators becomes clear, we must address why cells require multiple regulators with similar functions and how they cooperate to create diverse protrusions. We characterized Diaphanous (Dia) and Enabled (Ena) as a model, using complementary approaches: cell culture, biophysical analysis, and *Drosophila* morphogenesis. We found that Dia and Ena have distinct biochemical properties that contribute to the different protrusion morphologies each induces. Dia is a more processive, faster elongator, paralleling the long, stable filopodia it induces in vivo, while Ena promotes filopodia with more dynamic changes in number, length, and lifetime. Acting together, Ena and Dia induce protrusions distinct from those induced by either alone, with Ena reducing Dia-driven protrusion length and number. Consistent with this, EnaEVH1 binds Dia directly and inhibits DiaFH1FH2-mediated nucleation in vitro. Finally, Ena rescues hemocyte migration defects caused by activated Dia.

## Highlights

• Dia and Ena differ biochemically, promoting distinct filopodia dynamics
• Dia and Ena colocalization negatively regulates filopodia
• Ena’s EVH1 binds Dia’s FH1 and reduces Dia-driven filopodia and actin nucleation
• Ena rescues DiaΔDAD inhibition of hemocyte migration speed to wounds in vivo

## Graphical Abstract

**Figure:**
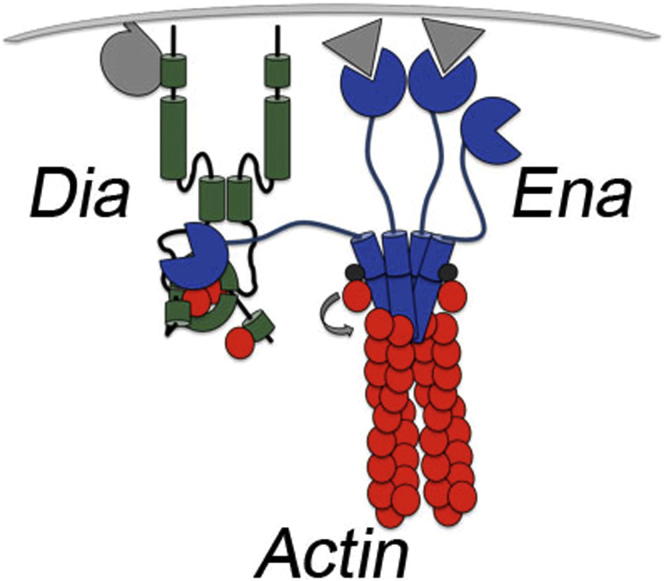

## Teaser

Bilancia et al. show that the actin regulators Dia and Ena differ biochemically and produce distinct protrusions. Dia is more processive and forms stable filopodia. Ena promotes dynamic filopodia. Dia and Ena directly bind each other, modulating their activities. Ena reduces Dia-driven filopodia, actin nucleation, and effects on cell migration.

## Introduction

Actin-based cell protrusions are a hallmark of migrating cells during development and disease. Migrating cells use two protrusion types: lamellipodia, broad protrusions supported by short-branched actin filaments, and filopodia, narrow processes containing parallel unbranched actin filaments. Filopodia are thought to be exploratory environment sensors, while lamellipodia provide the driving force for motility.

Key regulators shape the actin cytoskeletal architecture required for protrusions. Functions of individual actin regulators in vitro and in simple cell types are well studied, but how cells utilize different suites of actin regulators, some with similar functions, to make functionally distinct protrusions remains unclear. It is also unknown how the regulatory network is controlled by crosstalk among proteins to modify their activities and protrusion dynamics. We used two unbranched actin filament polymerases, Diaphanous (Dia) and Enabled (Ena), as a model to understand mechanistic differences between individual actin regulators with similar functions and how they work together to regulate actin dynamics and protrusions.

Dia is a Diaphanous-related formin (DRF), which nucleate and elongate unbranched actin filaments (Breitsprecher and Goode, 2013). *Drosophila* Dia plays many important roles in development, driving cellularization (Grosshans et al., 2005), regulating myosin, adhesion, and protrusive behavior during epithelial morphogenesis (Homem and Peifer, 2008, 2009), and controlling polarized epithelial secretion (Massarwa et al., 2009). Mammalian DRFs are also important actin regulators, controlling adhesion and cell protrusive behavior in culture (e.g., Yang et al., 2007; Gupton et al., 2007); via these roles they are implicated in human disease (DeWard et al., 2010).

**Figure 1:**
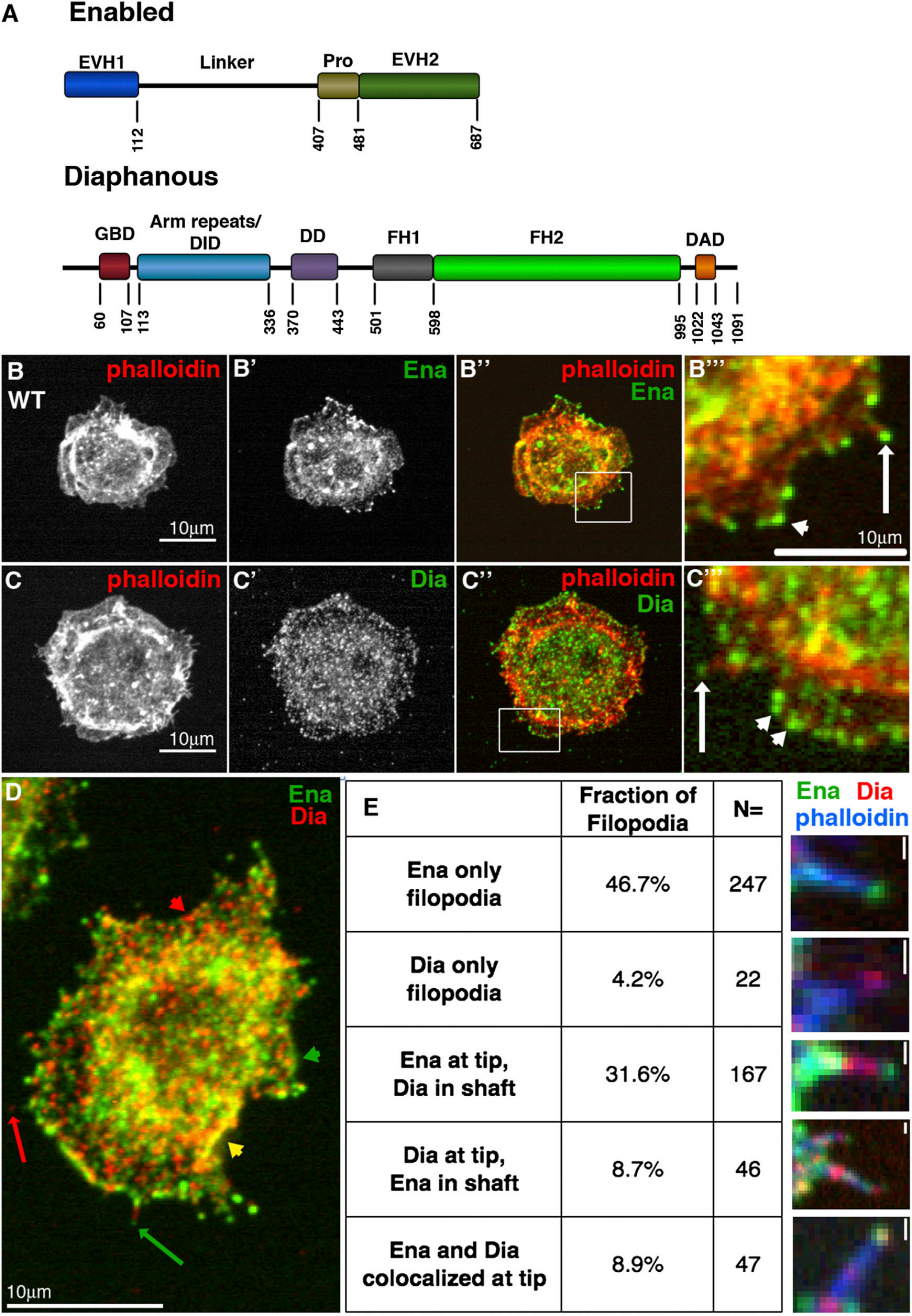
Endogenous Ena and Dia in D16 Cells (A) *Drosophila* Ena and Dia. (B–E) *Drosophila* D16 cells; arrows, filopodia; white arrowheads, lamellipodia. (B–C″′) Ena (B′–B″′) or Dia (C′–C″′) and phalloidin (B and C). (D) Ena and Dia. Dia-only filopodium (red arrow), Ena-only filopodium (green arrow), cortical Ena (green arrowhead), cortical Dia (red arrowhead), and cortical colocalization (yellow arrowhead). (E) Quantification of Ena and Dia localization in filopodia and representative images (right panels; scale bars, 0.5 μm).

DRFs share conserved domains (Figure 1A): the guanosine triphosphatase binding domain (GBD), Dia interacting domain (DID), dimerization domain, formin homology 1 and 2 (FH1 and FH2), and Dia autoinhibitory domain (DAD). DRFs are autoinhibited by association of the DAD and DID and activated when guanosine-triphosphate-bound Rho binds the GBD, releasing autoinhibition and allowing cortical recruitment (Alberts, 2001; Li and Higgs, 2003; Otomo et al., 2005; Rose et al., 2005; Gorelik et al., 2011). Once activated, the FH2 nucleates actin filaments (Pruyne et al., 2002; Sagot et al., 2002) and remains processively associated with barbed ends to promote monomer addition and block capping (Zigmond et al., 2003; Higashida et al., 2004; Romero et al., 2004; Kovar and Pollard, 2004). The FH1, a polyproline motif that binds profilin (Chang et al., 1997), increases barbed end elongation (Romero et al., 2004; Kovar et al., 2006).

DRF FH1 and FH2 domains cooperate to polymerize actin, making them targets for negative regulators. A wide range of proteins negatively regulate formins, e.g., yeast Bnr1’s FH2 is bound by Smy1 to slow elongation or by Bud14 to displace it from filaments (Chesarone et al., 2009; Chesarone-Cataldo et al., 2011). Diaphanous interacting protein binds mDia2 and can inhibit filopodia and actin assembly (Eisenmann et al., 2007), while Cip4 antagonizes Dia by inhibiting nucleation (Yan et al., 2013). Less is known about how multiple actin-binding proteins work together to regulate each other’s activity. WAVE and the Arp2/3 complex, primary players in branched actin networks, can interact with mDia2 to inhibit filopodia (Beli et al., 2008), suggesting important regulatory interactions between proteins responsible for opposing actin structures. However, the nature and role of interactions between proteins generating similar actin structures, like Dia and Ena/vasodilator-stimulated phosphoprotein (VASP), remain to be seen.

Ena/VASP proteins promote unbranched actin filament elongation by antagonizing Capping Protein (Bear et al., 2002; Barzik et al., 2005; Applewhite et al., 2007) and riding processively on barbed ends, promoting actin monomer addition (Breitsprecher et al., 2008; Hansen and Mullins, 2010). VASP also bundles actin filaments and may prevent Arp2/3-induced branching (reviewed in Bear and Gertler, 2009). Ena/VASP proteins, including the single *Drosophila* Ena/VASP, Ena (Gertler et al., 1990), share several conserved domains (Figure 1A). The Ena/VASP Homology 1 (EVH1) domain binds partners like Zyxin or Testin, often through a consensus FP4 motif (Phenylalanine and 4 Prolines). A Proline-rich region (Pro) recruits profilin-actin for barbed end addition. The EVH2 domain has G- and F-actin binding sites and a coiled-coil for tetramerization. Like Dia, Ena/VASP proteins regulate filopodia and lamellipodia in cell culture, and during development and disease (e.g., Gertler et al., 1996; Bear et al., 2002; Schirenbeck et al., 2006; Gates et al., 2007; Philippar et al., 2008).

Ena/VASP and Dia coimmunoprecipitate in flies, mice, and *Dictyostelium* (Grosse et al., 2003; Schirenbeck et al., 2005; Homem and Peifer, 2009). In *Drosophila* both localize at the leading edge of migrating epidermal cells, and their interplay modulates the function of each in vivo, as varying relative levels of Ena and Dia changes the protrusion profile during dorsal closure (Homem and Peifer, 2009). Thus, both Dia and Ena are important for shaping protrusions in vivo, but current data suggest they interact in complex ways to balance filopodia and lamellipodia during morphogenesis.

We explored how Dia and Ena regulate cell protrusions both individually and together, using cell biology and biophysical approaches. We found that Ena and Dia drive distinct protrusive behaviors that reflect differences in their processive actin filament assembly abilities. Ena and Dia directly bind through Ena’s EVH1 and Dia’s FH1 domains. When coexpressed, they induce protrusions distinct from those induced by either alone, and this seems largely explained by Ena’s EVH1 inhibiting Dia activity in vivo. Biophysical studies confirm that Ena’s EVH1 inhibits Dia nucleation but not elongation. Using *Drosophila*, we provide evidence that Ena modulates Dia activity and its effects on protrusive behavior in vivo during both dorsal closure and hemocyte migration.

## Results

### Dia and Ena Drive Distinct Filopodial Dynamics

Dia and Ena both promote unbranched processive actin filament elongation, leading us to ask why cells have two proteins performing similar functions. We hypothesized each has distinct properties, tailoring their activities to produce specific types of actin dynamics and cell protrusions. To test this, we characterized how they work individually to drive cell protrusions in culture. We used *Drosophila* D16 cells as a model, as they naturally form filopodia and lamellipodia (Figures 1B–1E). Furthermore, Dia is the single fly DRF and Ena the single Ena/VASP, eliminating redundancy. D16 cells express both Ena and Dia. Ena has a large cytoplasmic pool, but is enriched at the cortex, filopodia, and lamellipodia edges (Figures 1B–1B″′). Dia localizes similarly, with cytoplasmic staining and enrichment cortically and in filopodia (Figures 1C–1C″′). Surprisingly, despite similar roles in promoting filament elongation, only 9% of filopodia (n = 529; Figure 1E) had Ena and Dia colocalized at their tips. The most prominent class of filopodia contained only Ena (47%), while 32% had Ena at the tip and Dia in the shaft. Only a small fraction had Dia alone (4%) or Dia at the tip and Ena in the shaft (9%). Thus Ena seems dominant in D16 cells.

**Figure 2:**
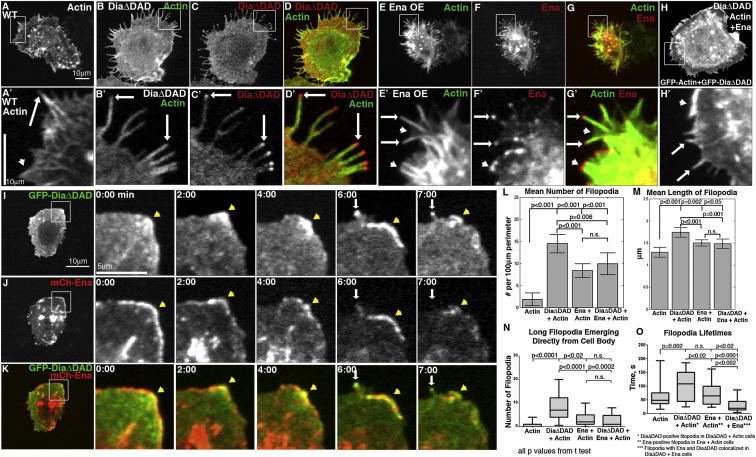
Ena and Activated Dia Coexpression Drives Protrusion Dynamics Distinct from Either Alone (A–O) D16 cells: arrows, filopodia; white arrowheads, lamellipodia; yellow arrowheads, Ena and Dia cortical colocalization. Transfection efficiency ranged from 10%–25% and expression levels were variable. Cells with midrange expression were used for all experiments. (A–H′) D16 cells (Movie S1) expressing GFP-actin (A and A′), GFP-DiaΔDAD+RFP-actin (B–D′), mCh-Ena+GFP-actin (E–G′), or GFP-DiaΔDAD+mCh-Ena+GFP-actin (H and H′). (I–K) Movie stills of GFP-DiaΔDAD (I and K) + mCh-Ena (J and K; Movie S3). Arrowhead, cortical colocalization in region without filopodia; white arrows, DiaΔDAD only filopodium. (L and M) Mean filopodia number (L) and length (M) for Actin (n = 16), DiaΔDAD (n = 34), Ena (n = 31), or DiaΔDAD+Ena (n = 28). Error bars = 95% confidence interval. (N) The 95^th^ percentile box and whisker plot, number of long filopodia (>1.5 μm) emerging from the cell body (actin, n = 11; DiaΔDAD, n = 30; Ena, n = 31; DiaΔDAD+Ena, n = 16). (O) Filopodia lifetimes (actin, n = 34; DiaΔDAD, n = 31; Ena, n = 33; DiaΔDAD+Ena, n = 14). See also Figure S1 and Movies S1, S3, and S4.

We next examined how each protein controls protrusion dynamics (Movie S1 available online). We expressed fluorescent actin alone (Figures 2A and 2A′), with GFP-DiaΔDAD (activated Dia lacking the DAD; Figures 2B–2D′ and S1F; expressed ∼30-fold over endogenous Dia), or with mCherry-Ena (mCh-Ena; Figures 2E–2G′ and S1F; expressed ∼3-fold over endogenous Ena). We hypothesized each would induce filopodia, but that number, length, or lifetime may differ. Consistent with this, DiaΔDAD (Figures 2B and 2B′) and Ena (Figures 2E and 2E′; Movie S1) drove ectopic filopodia and localized to filopodia tips (Figures 2D′ and 2G′). To determine if these filopodia differ, we quantified cell protrusions using a novel computational method, CellGeo (Tsygankov et al., 2014). CellGeo automatically identifies and tracks cell protrusions using a tree-graph representation of cell shape, allowing users to set mathematically precise definitions of filopodia and broad protrusions and to track and quantify them over time (Supplemental Experimental Procedures).

As expected, both DiaΔDAD and Ena significantly increased mean filopodial number and length relative to actin-only controls (Figures 2L and 2M). However, DiaΔDAD protrusion morphology and dynamics differed significantly from those driven by Ena. In DiaΔDAD, long filopodia (>1.5 μm) often emerged directly from the cell body (Figures 2B and 2B′; 8.1 filopodia/cell [n = 30] versus 0.9 filopodia/cell for actin-only [n = 11], Figure 2N), and the filopodia produced were strikingly stable (mean lifetime = 97 s versus 59 s for wild-type; Figure 2O and Movie S1). In contrast, Ena-driven filopodia were seen to emerge from fan-like broad protrusions by a process resembling convergent elongation, and multiple filopodia merged into fans (Figure 2G′; 2.7 events/movie, n = 18 movies; fans with Ena at the edge were rare in wild-type cells, being observed in 2/62 fixed cells stained with Ena). These “fans” had linear actin structures extending into the cell body, in contrast to wild-type cells (Figure 2A′ versus Figure 2E′). Ena also stimulated long filopodia emerging from the cell body (3.1/cell, n = 31; Figure 2N), but not as effectively as Dia. Ena-driven filopodia had a mean lifetime comparable to wild-type (68 s; Figure 2O). Thus, while Dia and Ena both elongate unbranched actin, they drive filopodia with distinct morphology and dynamics. This suggests their roles in filopodia are not redundant, but that each plays a distinct role as different cells create unique protrusion profiles.

### Different Actin Assembly Properties of Dia and Ena Might Underlie Their Ability to Drive Protrusions with Distinct Morphology and Dynamics

**Figure 3:**
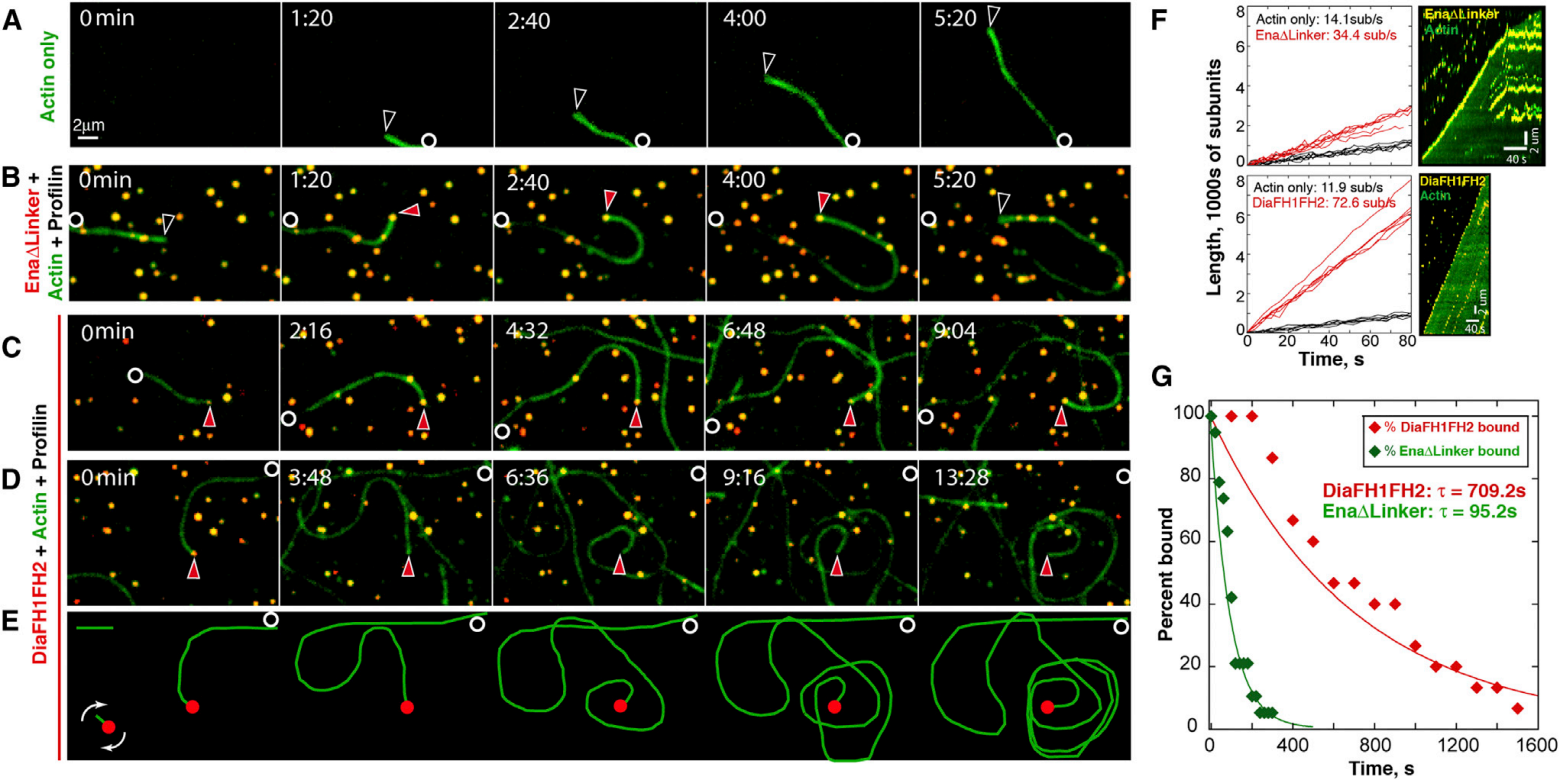
Dia Is a Faster Elongator and Is More Processive than Ena (A–D) TIRF montages, Movie S2: Oregon-green-labeled actin alone (A). Actin and *Drosophila* profilin with QD-biotin-SNAP-EnaΔLinker (B) or QD-biotin-SNAP-DiaFH1FH2 (C and D). Circles, filament pointed end; arrows, free filament barbed ends (open) or with EnaΔLinker or DiaFH1FH2 (red). QD blinks off in (D), but DiaFH1FH2 is present. (E) Filament in (D) traced (green); QD (red). (F) Filament elongation rates for controls (QD-free, black), EnaΔLinker (top left, red), or DiaFH1FH2 (bottom left, red). Representative kymographs (right) show single filaments with EnaΔLinker (top) or DiaFH1FH2 (bottom) processively bound to barbed end. Scale bars represent 2 μm (vertical) and 40 s (horizontal). (G) Single exponential fit of percent bound versus time gives mean residence time (τ) for DiaFH1FH2 (red) and EnaΔLinker (green). See also Figure S2 and Movie S2.

We hypothesized that the different biochemical properties of Dia and Ena account for the distinct protrusions they drive in vivo. To test this, we purified derivatives of *Drosophila* Dia and Ena and tested their actin assembly ability. As expected, Dia’s FH1FH2 domains (DiaFH1FH2) stimulated rapid pyrene actin assembly (Figures 5F, 6A, and S4D). Total internal reflection fluorescence microscopy (TIRF) with Oregon-green-labeled actin and quantum-dot-clustered (QD-clustered) DiaFH1FH2 revealed that DiaFH1FH2 accelerates actin filament elongation in the presence of profilin (Figures 3C–3E; Movie S2), relative to actin-only controls (Figure 3A; Movie S2; Romero et al., 2004; Jaiswal et al., 2013; Yan et al., 2013). DiaFH1FH2 rides processively on filament barbed ends, increasing the elongation rate ∼6-fold to 72.6 subunits/s versus 11.9 subunits/s for actin only (Figure 3F). We assessed Dia’s processivity by calculating the barbed end residence time of DiaFH1FH2, which averaged 709 s (Figure 3G). This would allow Dia to add ∼50,000 subunits/association, making it a very processive and efficient filament elongator, comparable to other DRFs (Romero et al., 2004; Kovar and Pollard, 2004; Kovar et al., 2006; Neidt et al., 2008).

We next examined Ena’s biochemical properties. We used an Ena derivative lacking the poorly conserved Linker (EnaΔLinker) because it was more stable than full-length Ena and stimulated comparable actin assembly (Figures S2A and S2B). Two-color TIRF of actin and QD-clustered EnaΔLinker revealed that Ena binds and rides processively on actin filament barbed ends (Figures 3B, 3F, and 3G; Movie S2), increasing the elongation rate 2.4-fold to 34.4 subunits/s (14.1 subunits/s for actin only; Figure 3F). We calculated the barbed end residence time for EnaΔLinker as 95.2 s (Figure 3G), yielding ∼3,200 subunits/association. Thus, both Dia and Ena promote actin filament elongation, but Dia remains processively associated with barbed ends ∼7-fold longer (709.2 s versus 95.2 s) and elongates them ∼2-fold faster (72.6 versus 34.4 subunits/s). These differences may help explain the distinct filopodial morphology and dynamics we observed. Dia induced longer, more persistent filopodia, while Ena stimulated shorter filopodia with wild-type lifetimes (Figures 2M–2O). In this model, once DiaΔDAD binds a barbed end, it is highly processive and quickly elongates filaments, resulting in long, stable filopodia. In contrast, Ena is less processive, which might make filaments susceptible to other actin regulators, resulting in more dynamic changes in filopodia number, length, and lifetime.

### Dia and Ena Together Produce Protrusions Distinct from Those They Induce Separately

Our previous work in embryos suggests Dia and Ena interact in a complex way to balance filopodia and lamellipodia (Homem and Peifer, 2009). To identify the mechanism by which they cooperate, we coexpressed DiaΔDAD and Ena in D16 cells. Strikingly, double overexpression (Figures 2H and 2H′; Movie S1) produced protrusions with morphology and dynamics distinct from those induced by Ena (Figures 2E and 2E′) or DiaΔDAD (Figures 2B and 2B′) alone. Morphologically, filopodia appeared thicker than wild-type but shorter than filopodia in DiaΔDAD cells (Figures 2A′, 2B′, and 2H′). Furthermore, while there were some broad protrusions, the fan-like regions of apparent convergent elongation induced by Ena alone were strikingly reduced (0.9/movie in DiaΔDad+Ena [n = 11] versus 2.7/movie for Ena alone; Figures 2H and 2H′ versus Figures 2E and 2E′). Coexpressing Ena and DiaΔDAD reduced mean filopodia number and length relative to DiaΔDAD alone (Figures 2L and 2M). Filopodia lifetimes are also reduced relative to DiaΔDAD or Ena cells alone (Figure 2O). There was also a striking effect on the number of long filopodia (>1.5 μm) emerging directly from the cell body, which was reduced from 8.1/cell to 2.25/cell (n = 16; Figure 2N). Ena does not need to be highly overexpressed relative to DiaΔDAD to have this effect (Figure S1F). These results are consistent with work in *Drosophila* embryos, where Ena coexpression reduced DiaΔDAD-driven filopodia number (Homem and Peifer, 2009). Thus, when coexpressed, Ena reduces filopodia number and length induced by active Dia, consistent with a model where Ena’s interaction with Dia is part of a negative regulatory mechanism.

### When Ena and Active Dia Colocalize, Filopodia Retract

D16 cells have cortical regions where endogenous Dia and Ena colocalize and other areas where only Ena or Dia localize (Figure 1D). Most filopodia are dominated by Ena, and they only occasionally colocalized at filopodial tips (Figures 1D and 1E). Our differential function hypothesis predicts that structures where they colocalize will exhibit different dynamics from those with only one or the other. We tested this by coexpressing GFP-DiaΔDAD and mCh-Ena and observing protrusion dynamics when they colocalize. As we saw in fixed cells (Figures 1D and 1E), most filopodia had DiaΔDAD or Ena alone (94% of 539 filopodia; Figures 2I–2K; Movie S3), and strong cortical colocalization correlated with regions of few filopodia. This is consistent with quantification showing a reduction in filopodia number by coexpressing DiaΔDAD and Ena, relative to DiaΔDAD alone (Figure 2L).

A small fraction of filopodia (6% of 539 filopodia) had strong DiaΔDAD and Ena colocalization (Figures S1A–S1C; Movie S4). Quantification revealed that Ena and DiaΔDAD colocalized on these filopodia tips for an average of 20 s, shorter than their individual tip residence times (DiaΔDAD = 95 s; Ena = 56 s; Figure S1E). After colocalization, most filopodia retracted (67%), folded back into the cortex (12%), or stalled (3%) (Figure S1D). These data are consistent with quantification of filopodia length, which is reduced by DiaΔDAD and Ena coexpression (Figure 2M). This is strikingly different from DiaΔDAD-only filopodia in the same cells, with mean lifetimes ≥190 s, supporting the idea that Dia and Ena can act separately or together to control distinct protrusion dynamics.

### Dia and Ena Directly Interact through Ena’s EVH1 and Dia’s FH1 domains

**Figure 4:**
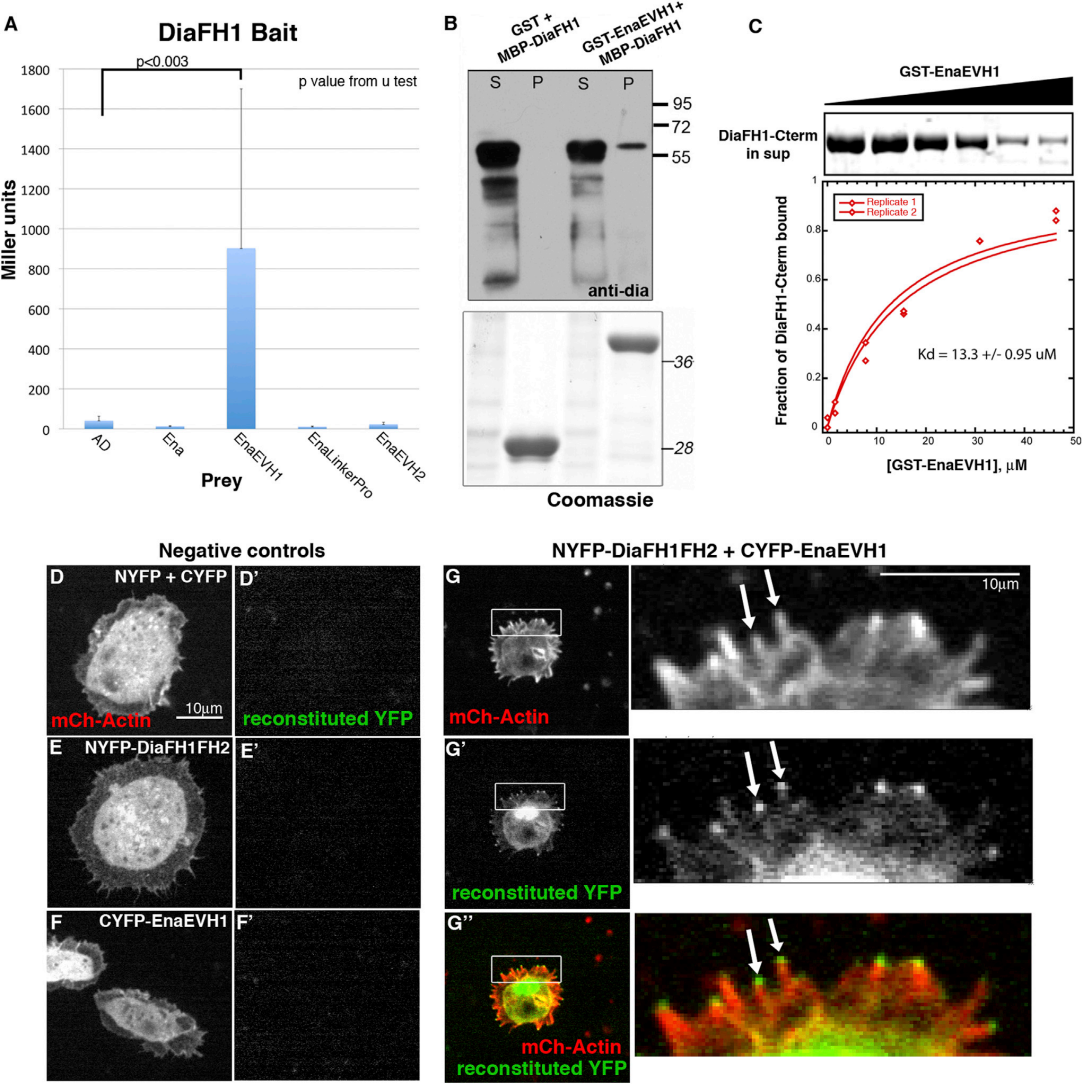
Ena and Dia Directly Bind and Interact in D16 Cells (A) Yeast two-hybrid βgal assays with DiaFH1 bait. Mean Miller units ± SD. (B) Top: GST-EnaEVH1 pulls down MBP-DiaFH1; S, supernatant; p, pellet. Bottom: Coomassie verifying equal loading. (C) Purified DiaFH1-Cterm is pulled down by GST-EnaEVH1. Top: Coomassie stained gel of DiaFH1-Cterm recruitment from supernatant with increasing concentrations of GST-EnaEVH1. Bottom: plot of dependence of DiaFH1-Cterm bound over a range of GST-EnaEVH1 concentrations. Average equilibrium dissociation constant = 13.3 μM. (D–G″) Split YFP in D16 cells. mCh-Actin (D, E, F, G) and reconstituted YFP fluorescence (D′, E′, F′, and G′) in NYFP+CYFP (D and D′), NYFP-DiaFH1FH2 alone (E and E′), CYFP-EnaEVH1 alone (F and F′), and NYFP-DiaFH1FH2+CYFP-EnaEVH1 (G–G″). Arrows in inset, YFP at filopodia tips. See Table S1.

Our data are consistent with the hypothesis that Ena negatively regulates Dia with important consequences for filopodia dynamics. To define mechanisms by which this occurs, we explored whether their colocalization and coimmunoprecipitation reflect indirect or direct interactions. We found that Ena’s EVH1 interacts with Dia’s FH1 domain in both yeast two-hybrid (Figure 4A; Table S1) and glutathione S-transferase (GST) pull-down assays (Figures 4B and 4C). DiaFH1-carboxyl-terminus (Cterm) binds EnaEVH1 with an equilibrium dissociation constant of 13.3 μM, consistent with a physiologically relevant interaction and similar to Ena/VASP EVH1 affinity for ActA (Holtzman et al., 2007). We next tested whether Ena and Dia interact in D16 cells, using split yellow fluorescent protein (YFP) bimolecular fluorescence complementation, in which the two halves of YFP, which are not individually fluorescent, reconstitute fluorescence if fused to proteins that bring them into close proximity (Kerppola, 2008; Gohl et al., 2010). We tagged DiaFH1FH2 with the N-terminal region of YFP (NYFP) and EnaEVH1 with the C-terminal region (CYFP). NYFP+CYFP does not reconstitute fluorescence (Figures 4D and 4D′), and neither NYFP-DiaFH1FH2 (Figures 4E and 4E′) nor CYFP-EnaEVH1 (Figures 4F and 4F′) fluoresces alone. However, coexpressing NYFP-DiaFH1FH2 and CYFP-EnaEVH1 resulted in YFP fluorescence internally and at filopodia tips (Figures 4G–4G″). These data confirm that EnaEVH1 and DiaFH1FH2 come into close proximity in cells and, with the data above, suggest direct Ena:Dia binding is important for regulating cell protrusions.

### EnaEVH1 Is Sufficient to Reduce Dia-Driven Actin Dynamics

**Figure 5:**
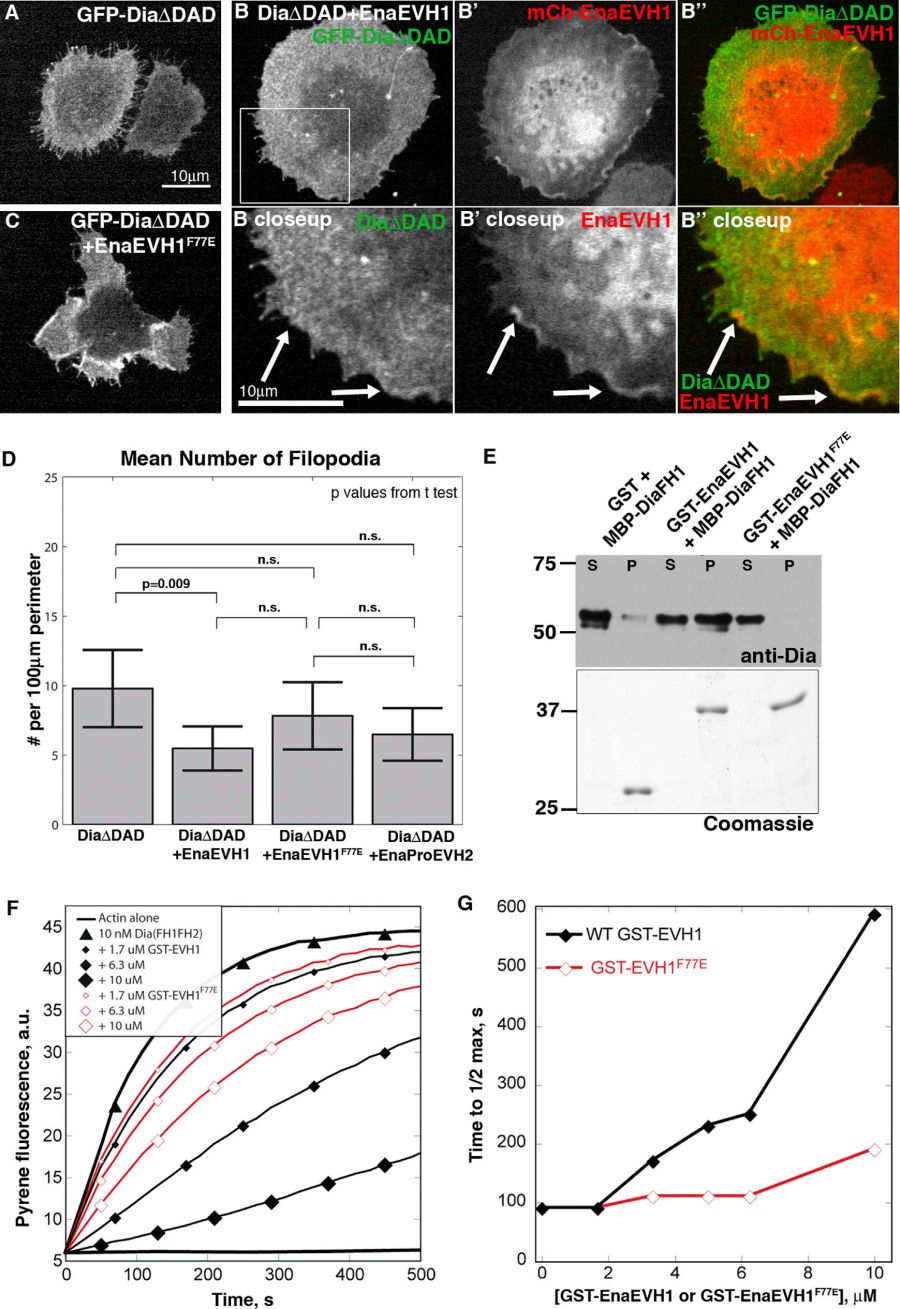
EnaEVH1 Is Sufficient to Reduce Dia-Driven Filopodia (A–C) D16 cells with GFP-DiaΔDAD alone (A), GFP-DiaΔDAD (B, B″, and B closeup) + mCh-EnaEVH1 (B′, B″, and B′ closeup), or GFP-DiaΔDAD+mCh-EnaEVH1^F77E^ (C). Arrows, cortical EnaEVH1. (D) Mean filopodia number, DiaΔDAD alone (n = 27), DiaΔDAD+EnaEVH1 (n = 28), DiaΔDAD+EnaEVH1^F77E^ (n = 26), and GFP-DiaΔDAD+EnaProEVH2 (n = 29); error bars = 95% confidence interval. (E) GST pull-down of DiaFH1 by GST, GST-EnaEVH1, or GST-EnaEVH1^F77E^. S, supernatant; p, pellet. Bottom: Coomassie verifying equal load. (F) Pyrene actin assembly with profilin and 10 nM DiaFH1FH2 (triangles), plus GST-EnaEVH1 (black diamonds) or GST-EnaEVH1^F77E^ (red diamonds). (G) Time it takes 10 nM DiaFH1FH2 to stimulate 1/2 max steady-state pyrene fluorescence (maximum actin assembly) versus concentration of GST-EVH1 constructs. See Figure S3.

Ena and DiaΔDAD coexpression reduces filopodia number and length, and their colocalization correlates with low filopodia number or retraction (Figures 2 and S1D). We hypothesized that direct EnaEVH1:Dia binding allows Ena to modulate Dia activity. To test this, we coexpressed GFP-DiaΔDAD (Figures 5B and 5B″) with mCh-EnaEVH1 (Figures 5B′ and 5B″) in D16 cells, comparing them to DiaΔDAD-only cells (Figure 5A). EnaEVH1 expression is sufficient to significantly reduce the number of DiaΔDAD-induced filopodia (Figure 5D). Consistent with this, although full-length Ena significantly reduced filopodia induced by DiaΔDad (Figure 2L), EnaProEVH2, lacking the EVH1 domain, did not do so (Figure 5D).

We next took this exploration in vitro. EnaΔLinker inhibits stimulation of actin assembly by DiaFH1FH2 in pyrene assays (Figure S3A), consistent with reduced filopodia induction in cells. We next tested if EnaEVH1 is sufficient to alter Dia activity, by performing actin assembly assays with DiaFH1FH2 and profilin with or without EnaEVH1. Bulk assays showed that EnaEVH1 has no effect on spontaneous actin assembly (Figures S4A and S4B) but inhibits stimulation of actin polymerization by DiaFH1FH2 (Figures 5F and 5G). Thus, EnaEVH1 alone is sufficient to reduce Dia-driven actin dynamics in vitro and in cell culture.

To test if Dia inhibition requires direct binding via EnaEVH1, we used the EVH1 domain crystal structure (Prehoda et al., 1999; Ball et al., 2000) to design mutants predicted to reduce ligand binding. We mutated the canonical ligand-binding phenylalanine 77 to glutamic acid to create the EnaEVH1^F77E^ mutant. GST pull-downs with EnaEVH1^F77E^ showed reduced binding to DiaFH1 (Figure 5E), suggesting EnaEVH1 binding requires the canonical ligand-binding site. We tested if the EnaEVH1^F77E^ mutation reduced Ena’s ability to inhibit Dia-driven actin dynamics, coexpressing GFP-DiaΔDAD and mCh-EnaEVH1^F77E^ in D16 cells (Figure 5C). Unlike EnaEVH1, EnaEVH1^F77E^ did not significantly reduce mean filopodia number induced by DiaΔDAD (Figure 5D). We also examined the effect of EnaEVH1^F77E^ in pyrene assays, assessing whether direct association is required for EnaEVH1 to reduce Dia-mediated actin assembly in vitro. Consistent with cell experiments, EnaEVH1^F77E^ had a significantly reduced ability to inhibit DiaFH1FH2 actin assembly (Figures 5F and 5G). Taken together, these data show that EnaEVH1 is sufficient to negatively regulate Dia and suggest that it acts through canonical EnaEVH1 ligand-binding residues. Our functional assays also suggest the possibility that EnaEVH1^F77E^ reduces, but does not eliminate, Ena:Dia interactions and thus acts as a hypomorph. Our data support a model where negative regulation of Dia by direct binding of EnaEVH1 is part of the complex mechanism regulating actin assembly and cell protrusions. Ena and Dia also may affect one another by additional mechanisms, such as competition for barbed ends. Consistent with this, EnaProEVH2 also can reduce actin assembly by DiaFH1FH2 in pyrene assays (Figures S3B and S3C).

### Ena’s EVH1 Domain Inhibits Dia-Mediated Nucleation

**Figure 6:**
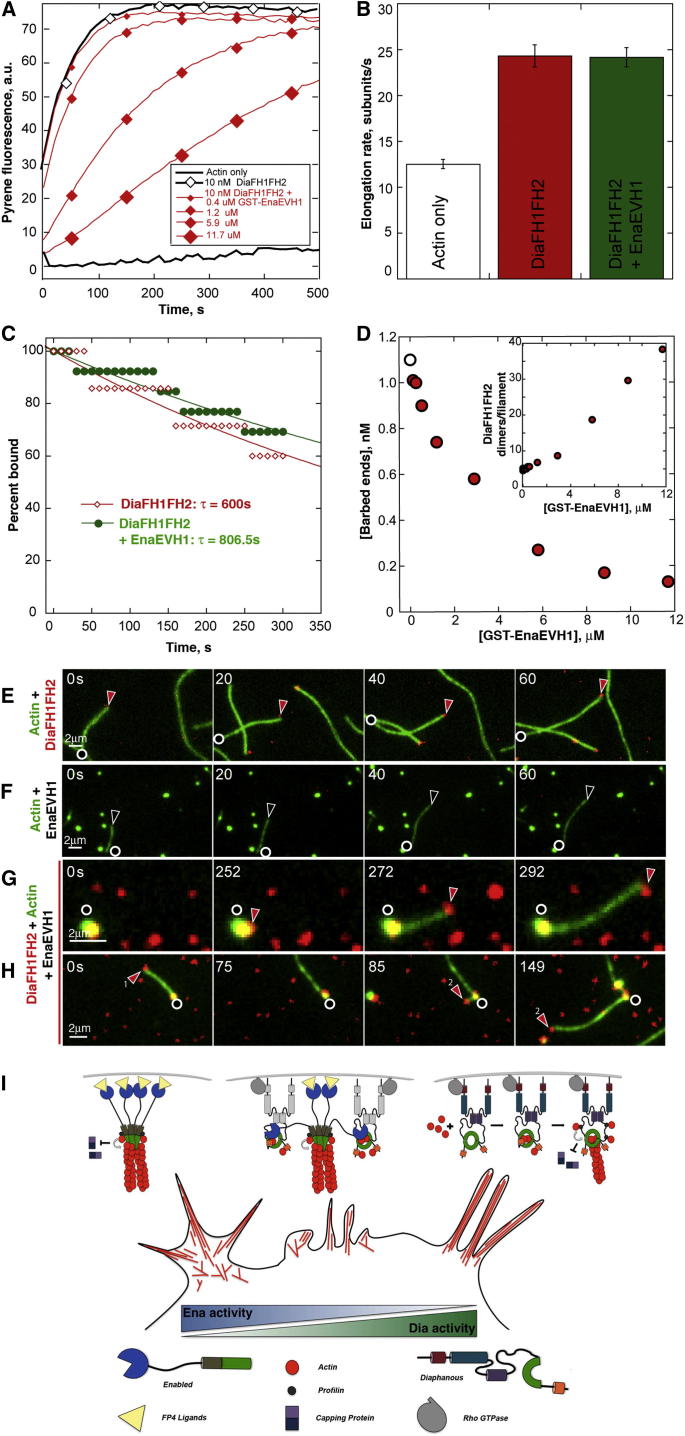
EnaEVH1 Inhibits Dia Nucleation All assays without profilin. (A) Pyrene actin assembly; 10 nM DiaFH1FH2+increasing GST-EnaEVH1. (B) Actin elongation rates calculated from TIRF. Actin alone (white), actin+DiaFH1FH2 (red), or actin+DiaFH1FH2+GST-EnaEVH1(green); n = 2; error bars = ±SEM. (C) Percent of barbed ends remaining bound to SNAP-549-DiaFH1FH2 in absence (red) or presence (green) of 5 μM GST-EnaEVH1. Single exponential fits show mean residence time (τ). (D) DiaFH1FH2 nucleation calculated from pyrene assays in (A). Concentration of barbed ends nucleated by 10 nM DiaFH1FH2 in the absence (white) or increasing concentrations of GST-EnaEVH1 (red). Inset: mean number of DiaFH1FH2 dimers required to nucleate a filament in the absence or presence of increasing GST-EnaEVH1. (E–H) TIRF montages: 1.5 μM Oregon-green actin with SNAP-549-DiaFH1FH2 (red) (E), GST-EnaEVH1 (F), or GST-EnaEVH1 and SNAP-549-DiaFH1FH2 (G and H) (Movie S5); circles, filament pointed end; arrows, free filament barbed ends (open) or with DiaFH1FH2 (red). (I) Model of Ena inhibition of Dia and effects on protrusions. See also Figure S4 and Movie S5.

Our data reveal that EnaEVH1 can inhibit Dia function by direct binding, but how it inhibits actin assembly remained unclear. DiaFH1 also binds to profilin-actin, which is the rate-limiting step of formin-mediated barbed end elongation (Vavylonis et al., 2006; Paul and Pollard, 2008). Therefore, we hypothesized that EnaEVH1:DiaFH1 association interferes with elongation by disrupting profilin binding to DiaFH1. To test this, we repeated actin assembly assays with DiaFH1FH2 and EnaEVH1 without profilin, but found that EnaEVH1 still inhibited DiaFH1FH2 (Figures 6A and S4G). Thus, blocking profilin is not the main role of EnaEVH1 binding.

To further probe mechanism, we performed TIRF, using Oregon-green-labeled actin and red-labeled SNAP-549-DiaFH1FH2 to assess actin filament elongation in the presence and absence of EnaEVH1. In the absence of profilin, DiaFH1FH2 (Figure 6E; Movie S5) increased barbed end actin filament elongation from 12.5 to 24.3 subunits/s (Figure 6B; this was surprising since other formins slow elongation in the absence of profilin, and it will need to be explored further) and had a mean residence time of 600 s (Figure 6C). EnaEVH1 alone caused actin puncta formation (Figure 6F; Movie S5), but this had little effect on actin assembly (Figures S4A and S4B). EnaEVH1 did not alter the DiaFH1FH2 elongation rate (24.2 subunits/s; Figure 6B), residence time (806.5 s; Figure 6C), or its effect in seeded actin assembly assays (Figures S4D and S4E), showing that EnaEVH1 does not inhibit Dia’s ability to processively elongate actin filaments. Similarly, EnaΔLinker did not alter Dia’s elongation rate (Figures S4C and S4F).

We next tested whether EnaEVH1 inhibits actin nucleation by Dia. Knowing the barbed end elongation rate from TIRF allowed us to calculate barbed end concentrations from pyrene actin assembly assays (Figures 6A, 6D, and S4H; as in Higgs et al., 1999). We found that increasing concentrations of EnaEVH1 significantly reduced the concentration of DiaFH1FH2-nucleated barbed ends (e.g., 1.0 nM without EnaEVH1 to 0.27 nM at 5.8 μM EnaEVH1; Figures 6D and S4H). These data suggest that only 20% of DiaFH1FH2 dimers nucleate a new filament under these conditions. Thus, EnaEVH1 reduces the nucleation efficiency of DiaFH1FH2. TIRF revealed that EnaEVH1 recruits DiaFH1FH2 to actin puncta; most do not initiate actin assembly, but occasionally Dia-associated barbed ends elongated away from these puncta (Figures 6G and 6H; Movie S5), suggesting that Dia can escape inhibition and initiate actin assembly. These data, together with the lack of change in elongation, support a model where EnaEVH1 binds DiaFH1 and actin to inhibit Dia nucleation (Figure 6I), but do not rule out the possibility that EnaEVH1 also interacts with the FH2 domain.

### Dia-Driven Protrusions Are More Dynamic in Areas of High Endogenous Ena during *Drosophila* Dorsal Closure

**Figure 7:**
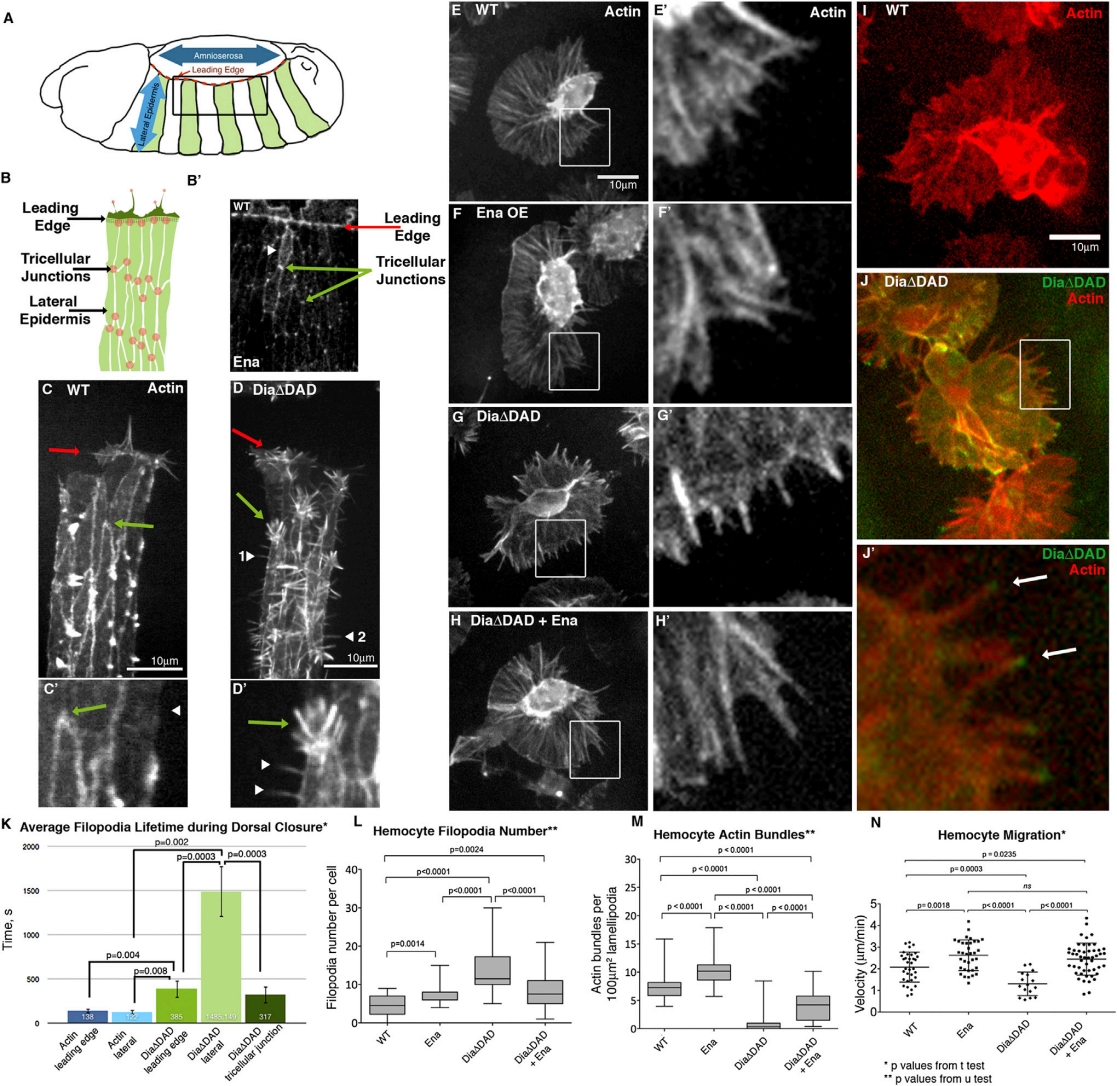
Ena Negatively Regulates Activated Dia during *Drosophila* Development (A) Dorsal closure; green stripes, *en*GAL4-driven Actin-expressing epidermis in (C)–(D′). (B) Single Actin-expressing stripe with leading edge protrusions (dark green), lateral epidermis (light green), and high Ena localization (red). (B′) Ena staining at leading edge (red arrow), lateral cell border (white arrowhead), and tricellular junctions (green arrows). (C–D′) Dorsal closure imaged by GFP-Actin for wild-type (C and C′) (Movie S6) and DiaΔDAD (D and D′) (Movie S7). Leading edge, red arrows; tricellular junctions, green arrows; lateral cell borders, white arrowheads. (E–H′) F-actin (LifeActGFP) in wild-type (E and E′), Ena (F and F′), DiaΔDAD (G and G′), and DiaΔDAD+Ena (H and H′) hemocytes. (I–J′) F-actin (mCh-Moesin) in wild-type (I) or GFP-DiaΔDAD-expressing (J and J′) hemocytes. DiaΔDAD at filopodia tips (arrows). (K) Mean filopodia lifetime: actin leading edge, n = 95; actin lateral, n = 28; DiaΔDAD leading edge, n = 140; DiaΔDAD lateral, n = 110; and DiaΔDAD tricellular junctions, n = 68. Error bars ± SEM. (L and M) Number of hemocyte filopodia and actin bundles: wild-type, n = 34; Ena, n = 37; DiaΔDAD, n = 36; DiaΔDAD+Ena, n = 38. Median and interquartile range. (N) Hemocyte migration speed: wild-type, n = 34; Ena, n = 35; DiaΔDAD, n = 16; DiaΔDAD+Ena, n = 50. Mean ± SD. See also Movies S6 and S7.

We next tested whether Ena plays the same negative regulatory role in the complex environment in vivo (Figure 7A). Ena and Dia shape the suite of protrusions formed during *Drosophila* dorsal closure in vivo; notably, Ena coexpression reduced DiaΔDAD-induced filopodia, and reducing Ena activity increased DiaΔDAD-induced filopodia number and length (Homem and Peifer, 2009), consistent with our D16 cell results. To explore the role of endogenous Ena in regulating Dia-driven actin dynamics in vivo, we imaged wild-type embryos (Movie S6) and those expressing DiaΔDAD, which induced ectopic filopodia at all cell borders (Figures 7C and 7C′ versus Figures 7D and 7D′; Movie S7). We compared protrusion dynamics in areas of the cortex with low or high endogenous Ena levels (Figures 7B and 7B′), comparing the leading edge and tricellular junctions (high Ena) with lateral borders (low Ena). This revealed two distinct filopodia populations with different dynamics, depending on endogenous Ena levels. Strikingly, Dia-induced filopodia at lateral cell borders, where Ena levels were low, were long lived (Figure 7K) and emerged directly from the cell body (Figures 7D and 7D′, arrowheads), reminiscent of long, stable DiaΔDAD filopodia in D16 cells (Figure 2B′). In contrast, filopodia Dia induced from tricellular junctions (Figures 7D and 7D′, green arrows), areas with high endogenous Ena levels, were shorter lived (Figure 7K) and emerged from dynamic structures with both lamellipodial and filopodial character, thus resembling those at the leading edge where Ena levels are also high (Figure 7C, red arrow). These data are consistent with the hypothesis that Ena can alter Dia activity in vivo.

### Ena Rescues DiaΔDAD-Induced Defects in Filopodia Number, Actin Bundle Formation, and Migration Speed in Hemocytes

Dorsal closure is driven by a sheet of planar polarized adherent and collectively migrating epithelial cells, which are distinct from the D16 cells we used as a model. To test whether Ena:Dia interactions play a role in other tissues in vivo, we examined *Drosophila* hemocytes, immune cells roughly analogous to macrophages. These cells undergo stereotypical migration throughout the embryo and exhibit chemotactic migration to wounds (Wood and Jacinto, 2007). Ena promotes filopodia number and length, lamellipodial dynamics, and migration speed in hemocytes (Tucker et al., 2011). However, Dia’s role and interaction with Ena remained unclear.

We thus examined whether DiaΔDAD can promote filopodia in hemocytes and assessed whether Ena can negatively regulate that activity. We analyzed inflammatory recruitment of hemocytes on the ventral side of stage 15 embryos, comparing wild-type (Figures 7E, 7E′, and 7I), Ena overexpression (Figures 7F and 7F′), DiaΔDAD (Figures 7G, 7G′, 7J, and 7J′), and DiaΔDAD+Ena (Figures 7H and 7H′) hemocytes. Ena overexpression increased filopodia number and migration speed to wounds (Figures 7L and 7N). Ena also increases actin bundles in hemocyte lamellipodia (Figure 7M). DiaΔDAD localized to filopodia tips (Figures 7J and 7J′) and increased filopodia number more effectively than Ena (Figure 7L), but those filopodia lacked the actin bundles induced by Ena.

This in vivo tissue also allowed us to assess the functional consequences of manipulating Ena and Dia activity. Strikingly, while Ena expression enhanced migration velocity, activated Dia reduced it (Figure 7N). Thus, increasing filopodia number alone cannot enhance migration speed, suggesting that Ena-induced bundled actin architecture in lamellipodia might be an important driver of hemocyte migration. Finally, we examined whether coexpressing Ena was sufficient to rescue the DiaΔDAD phenotypes. Ena coexpression reduced filopodia number to Ena-only levels (Figure 7L), matching our D16 cell results (Figure 2L). Inflammatory migration speed was also rescued, with DiaΔDAD+Ena hemocytes migrating at speeds similar to those overexpressing Ena alone (Figure 7N). Surprisingly, while actin bundles were significantly increased in DiaΔDAD+Ena cells, they did not reach wild-type or Ena-only numbers (Figure 7M), suggesting that a few actin bundles are sufficient to drive migration or that they only function minimally to promote migration speed. Together with our dorsal closure work, these data support the idea that Ena can negatively regulate Dia in vivo to control cell protrusions and migration during morphogenesis.

## Discussion

As actin regulator functions become clearer, we must address how they work in parallel or together in vivo. Ena and Dia provide a superb model; both are key actin regulators that facilitate processive unbranched actin filament assembly, and our work in vivo suggests they work together to promote protrusions during embryogenesis via a complex mechanism. We used an interdisciplinary approach to explore how Ena and Dia’s biochemical properties and direct interaction shape their effects on actin dynamics and cell behavior in vivo.

Since Ena and Dia both promote unbranched actin polymerization, we first asked why cells use two similar machines. We found both Ena and Dia promote filopodia in cell culture, but Ena- and Dia-driven filopodia had substantially different morphology and dynamics. Our data suggest these differences reflect distinct biochemical properties. Dia is a faster and more processive elongater than Ena, helping explain why Dia-based filopodia are more persistent and Ena-based protrusions more dynamic. Ena and Dia may also elongate filaments nucleated by different proteins (e.g., Ena elongating Arp2/3 complex-initiated filaments and Dia elongating filaments it nucleated itself; Chesarone and Goode, 2009). Tuning the balance of Ena and Dia activity helps cells produce different suites of protrusions and diverse cell behaviors (Figure 6I).

We next examined how Ena and Dia work together. Our data are consistent with a model in which cells modulate Dia activity through negative regulation by Ena. EnaEVH1 binds to and inhibits Dia actin assembly in vitro. Inhibition occurs in the absence of profilin, and Dia’s elongation rate and processivity are not affected by EnaEVH1 or EnaΔLinker. Instead, we found EnaEVH1 inhibits DiaFH1FH2 nucleation. As VASP’s EVH1 binds mDia2’s FH2 (F. Gertler, personal communication), this might be a conserved mechanism for inhibiting formins. Since both Ena’s EVH1 and Dia’s FH1 domains have other partners that are essential for their functions, it will be important to generate mutants specifically blocking Ena:Dia interaction to further test these hypotheses.

How does EnaEVH1 binding inhibit actin nucleation by Dia? Several “stepping models” of formin actin assembly all share a role for conformational changes in the FH2 domain and actin (Paul and Pollard, 2009). One attractive but speculative hypothesis is that EnaEVH1:DiaFH1 binding inhibits conformational changes needed for nucleation and initiation of processive elongation. Indeed, the plant formin AFH1’s FH1 domain has a profilin-independent effect on barbed end elongation, likely by affecting FH2 domain conformation (Michelot et al., 2005). Actin may also play a role as DiaFH1FH2 is recruited to EnaEVH1-induced actin puncta seen in our TIRF assays, suggesting that EnaEVH1-actin association might stabilize Dia binding or help block nucleation. It will be important to examine how all three proteins interact to regulate Dia activity as part of a broader effort to determine mechanisms by which Ena inhibits Dia.

How does Ena regulation of Dia control cell protrusions? In TIRF, we observed that DiaFH1FH2 accumulated at EnaEVH1-dependent actin puncta, but could escape and elongate filaments (Figures 6G and 6H). Such an inhibitory mechanism might allow quick modulation of active Dia, allowing it to be paused and released to promote actin nucleation and long, stable filopodia without multiple rounds of autoinhibition and cortical localization. Second, actin and nucleation promoting factors (NPFs) can bind formin DADs to enhance actin assembly (Moseley et al., 2004; Okada et al., 2010; Gould et al., 2011; Graziano et al., 2011, 2013; Heimsath and Higgs, 2012; Breitsprecher et al., 2012; Jaiswal et al., 2013). Ena inhibition might counterbalance this mechanism by blocking Dia nucleation or interfering with the “rocket launcher” mechanism. Examining whether the DAD domain also modulates interactions among Ena, Dia, and actin will be important to further elucidate this negative regulatory interaction.

Our studies provide a foundation for future work, both in vitro and in vivo. For example, studying Ena and Dia with NPFs in vitro will be crucial to understanding mechanisms controlling the broad network of actin regulators. It will also be important to expand this work in vivo. Our mechanistic data support a model in which Ena and Dia play distinct roles when acting alone or together. In the simplest version of our model, Ena inhibits Dia, allowing cells to switch from long, persistent protrusions to a more dynamic mix of lamellipodia and filopodia. This fits well with our data in D16 cells and also helps explain what we observed in hemocytes in vivo; however, these may represent relatively simple systems, as our data and earlier work (Tucker et al., 2011; Tsygankov et al., 2014) suggest Ena plays the primary role in these cells. This model does not fully explain results observed in more complex tissues like leading edge cells during dorsal closure. In these cells, Ena and Dia are both required for the proper balance of filopodia and lamellipodia that ensures dorsal closure, and relative levels of Ena and Dia activity help regulate this balance (Gates et al., 2007; Homem and Peifer, 2009). Some features of leading edge cell behavior fit our simplest model, e.g., Ena overexpression reduces the number of filopodia induced by DiaΔDad and reducing Ena levels increases DiaΔDAD-induced filopodia, consistent with a negative regulatory role of Ena in vivo (Homem and Peifer, 2009). However, in this complex environment we observed other effects not predicted by our simplest model, e.g., coexpressing Ena and DiaΔDad significantly increased lamellipodial area (Homem and Peifer, 2009). These complexities likely reflect the presence and activity of other players like the Arp2/3 complex, which may compete with Ena and Dia for a limiting pool of actin. Ena may also be channeled away from Dia and to the ends of Arp2/3 generated branches. It will be important to examine how Ena and Dia are integrated with other actin regulators during dorsal closure.

Dorsal closure also provides a place to examine mechanisms driving polarized protrusive behavior. The restriction of filopodia to the dorsal side of leading edge cells is due in part to limited Dia activation, as activated Dia induces filopodia on all surfaces of all epidermal cells. Our work suggests that the types of Dia-driven protrusions are regulated by the localization of endogenous Ena. At places with low cortical Ena like lateral cell borders, Dia induces long-lived filopodia emerging from the cell body. In contrast, at dorsal cell borders, where Ena is enriched, activated Dia induces a dynamic mix of lamellipodia and filopodia like those at the leading edge. These data are consistent with the idea that polarized Ena localization and localized Dia activation help regulate leading edge polarization and protrusion dynamics. It will be exciting to define mechanisms leading to this asymmetry.

## Experimental Procedures

### Cell Analysis

D16C3 cells were cultured in Schneider’s Media+FBS+insulin, transfected with FugeneHD, and imaged on glass-bottom dishes after 48–72 hr every 2 s for 2–6 min on a Wallac Ultraview Confocal. Expressing tagged proteins versus endogenous shows Ena is ∼3-fold overexpressed (24% transfected cells) and Dia is ∼30-fold overexpressed (16% transfected cells; Figure S1F). For fixed images, cells were plated on coverslips, fixed with 32% paraformaldehye solution (EM Sciences) diluted to 10% in PBS, and stained for Ena, Dia, or tetrarhodamine-isothiocyanate-phalloidin. Antibodies are in Supplemental Experimental Procedures. ImageJ (National Institutes of Health) was used to adjust brightness/contrast. We quantified ≥60 fr from 11–35 cells using *CellGeo* (number/length) or manually (lifetime/persistence). Filopodia definition was ≥1 μm long and <0.77 μm wide.

### Protein Purification

Dia or Ena were induced with 0.5 mM isopropylthio-β-galactoside (IPTG) for 16 hr at 16°C and purified from Talon Metal Affinity Resin. Ena was gel purified on S20010/300GL and Dia was dialyzed against formin buffer and stored at −80°C. SNAP tagging used SNAP-tag-T7-2(NEB) with a flexible linker (GGSGGS) between tag and start codon, and labeling was per manufacturer.

### TIRF

Images were collected every 2–4 s with an iXon electron-multiplying charge-coupled device (CCD) camera (Andor) on an Olympus IX-71 microscope with through-the-objective TIRF. Mg-ATP-actin (15% Oregon green) was mixed with 2XTIRF buffer and Ena or Dia ± 3.0 μM profilin, and imaged in a flow cell at 23°C. Biotinylated SNAP-tagged proteins were labeled with streptavidin-conjugated QDs. Ena or Dia were tracked manually for barbed end residence times. Filament elongation rates were calculated by measuring filament length over time in ImageJ. Nucleation was calculated as in Higgs et al. (1999). Curve fits and plots were generated with KaleidaGraph.

### Fluorescence Spectroscopy

Pyrene-actin fluorescence was measured with Safire^2^ fluorescent plate reader. The 10% pyrene-labeled Mg-ATP-actin monomer assembly was initiated by adding 50 mM KCl, 1 mM MgCl_2_, 1 mM EGTA, 10 mM imidazole pH 7.0, and Ena/Dia constructs.

### Yeast Two-Hybrid

Yeast two-hybrid used the LexA system and LacZ reporter strain EGY48. Constructs were tested pairwise for growth in selective media and in liquid β-galactosidase (βgal) assays. Bait constructs with activation domain alone were controls. Greater than or equal to three assays were performed per bait-prey pair.

### GST Pull-Down

N-terminally GST-tagged or maltose binding protein (MBP)-tagged proteins in BL-21 cells were induced by 0.5 mM IPTG and grown overnight at 18°C, and lysates were incubated with glutathione-Sepharose-4B for 2 hr at 4°C. Supernatants and bead eluates were analyzed by SDS-PAGE and Coomassie, or Dia immunoblot. For pull-downs with purified proteins, DiaFH1-Cterm and glutathione-Sepharose bead concentrations were kept constant and increasing amounts of GST-EVH1 were added, incubated for 20 min at 25°. Supernatants were analyzed by SDS-PAGE, and the bound faction of DiaFH1-Cterm was fit to a quadratic equation to give the equilibrium dissociation constant.

### *Drosophila*

Stocks are in Supplemental Experimental Procedures. Dorsal closure images were acquired every 5 s using 100X1.4NA PlanApoVC objective on a TE2000-E microscope (Nikon) with a VTHawk (VisiTech) and OrcaR2 CCD camera (Hammamatsu). GFP-expressing hemocytes images were acquired every 1 min for 1 hr postwounding on a spinning disc confocal (PerkinElmer). ImageJ was used for filopodia quantification and to track hemocytes. Hemocyte morphology, filopodia, and actin bundles were quantified from still images of LifeAct-expressing hemocytes.

### Statistical Analysis

Statistical comparisons were done by Student’s t test (Figures 2, 5, 7K, 7N, and S1) or Mann Whitney U test (Figures 4, 7L, and 7M).

## Supplemental Information

Document S1. Supplemental Experimental Procedures, Figures S1–S4, and Table S1

Movie S1, Related to Figure 2. Dia and Ena Induce Protrusions Distinct from One Another and from Those They Induce When Expressed Together Panel 1: Wild-type D16 cell expressing GFP-Actin has actin-based filopodia and lamellipodia. Panel 2: D16 cell expressing mCh-Actin (white) and GFP-DiaΔDAD (not shown) shifts cell protrusions to long, stable filopodia. Panel 3: D16 cell expressing GFP-Actin (white) and mCh-Ena (not shown) drives dynamic filopodia that often emerge from fan-like protrusions. Panel 4: D16 cell expressing GFP-Actin and GFP-DiaΔDAD (white) plus mCh-Ena (not shown). Coexpression produces filopodia that are longer than wild-type but shorter than DiaΔDAD cells, and fan-like protrusions seen in Ena cells are absent. Images (100× magnification) were taken every 2 s. Movie is displayed at 15 frames/s. Scale bar represents 10 μm. Time stamp is min:s.

Movie S2, Related to Figure 3. Spontaneous Actin Assembly Alone, in the Presence of Profilin and EnaΔLinker, or in the Presence of Profilin and DiaFH1FH2 Arrowheads mark filament barbed ends when unoccupied (open) or when occupied with EnaΔLinker or Dia (red). Top: TIRF movie of 1.5 μM actin (15% Oregon-green-labeled; green). Control for DiaFH1FH2 and EnaΔLinker. Middle: EnaΔLinker increases actin assembly. TIRF movie of 1.5 μM actin (15% Oregon-green-labeled; green), *Drosophila* profilin (Chickadee), and quantum dot-labeled biotin-SNAP-EnaΔLinker (red). Bottom: DiaFH1FH2 increases actin assembly. TIRF movie of 1.5 μM actin (15% Oregon-green-labeled; green), *Drosophila* profilin, and quantum dot-labeled biotin-SNAP-DiaFH1FH2 (red). Movie is displayed at 15 frames/s. Time is min:s.

Movie S3, Related to Figure 2. Strong Colocalization of DiaΔDAD and Ena at the Cell Cortex Results in Fewer Filopodia Time-lapse confocal microscopy movie showing GFP-DiaΔDAD (A, green) and mCh-Ena (B, red) colocalization at the cell cortex (merge, C), which corresponds with a low number of filopodia. One filopodium forms at the end of the movie, but only GFP-DiaΔDAD is present at the tip. Images (100× magnification) were taken every 2 s. Movie is displayed at 15 frames/s. Scale bar represents 1 μm.

Movie S4, Related to Figure 2. Colocalization of DiaΔDAD and Ena in Filopodia Often Results in Retraction Time-lapse confocal microscopy movie showing GFPDiaΔDAD (A, green) and mCh-Ena (B, red) in a filopodium. Colocalization (merge, C) is quickly followed by retraction of the filopodium. Images (100× magnification) were taken every 2 s. Movie is displayed at 15 frames/s. Scale bar represents 1 μm.

Movie S5, Related to Figure 6. Spontaneous Actin Assembly of DiaFH1FH2, EnaEVH1, or Both in the Absence of Profilin Top: Actin assembly is largely unaffected in the presence of EnaEVH1, although actin puncta form. TIRF movie of 1.5 μM actin (15% Oregon-green-labeled; green) + 5 μM GST-EnaEVH1 (unlabeled) in the absence of profilin. Movie shows actin puncta formation, although this has little effect on actin assembly in pyrene assays (see Figures S4A and S4B). Circle marks filament pointed end; arrowhead marks filament barbed end. Middle: DiaFH1FH2 actin assembly in the absence of profilin. TIRF movie of 1.5 μM actin (15% Oregon-green-labeled; green) + 1 nM SNAP-549-DiaFH1FH2 (red) in the absence of profilin. Circle marks filament pointed end; red arrowhead marks filament barbed end with DiaFH1FH2. Bottom: EnaEVH1 inhibits actin assembly by DiaFH1FH2. TIRF movie of 1.5 μM actin (15% Oregon-green-labeled; green) + 1 nM SNAP-549-Dia-FH1FH2 (red) + 5 μM GSTEnaEVH1 (unlabeled) in the absence of profilin. Movie shows green spots of actin accumulating in the presence of GST-EnaEVH1. Many of these colocalize with DiaFH1FH2 (red). White arrow marks EVH1/actin puncta. Eventually, a DiaFH1FH2 elongated filament emerges from the actin-DiaFH1FH2 puncta (red arrowheads). Movie is displayed at 15 frames/s. Time is min:s.

Movie S6, Related to Figure 7. Wild-Type *Drosophila* Embryonic Dorsal Closure Wild-type dorsal closure in an embryo expressing GFP-actin using *engrailed*-GAL4. Note endogenous Ena present. Control for Movie S7. Images (100× magnification) were collected every 5 s. Movie is displayed at 15 frames/s. Scale bar represents 10 μm. Time is hr:min:s.

Movie S7, Related to Figure 7. Dia-Driven Protrusions Are More Dynamic in Areas of High Ena Accumulation during *Drosophila* Embryonic Dorsal Closure Dorsal closure in an embryo expressing both GFP-actin and HA-DiaΔDAD using *engrailed*-GAL4. Endogenous Ena is enriched at the leading edge and at tricellular junctions relative to lateral cell borders. Images (100× magnification) were collected every 5 s. Movie is displayed at 15 frames/s. Scale bar represents 10 μm. Time is hr:min:s.
