# Supplementary material for: Enabled Negatively Regulates Diaphanous-Driven Actin Dynamics In Vitro and In Vivo
Source: Dev Cell. 2014 Feb 24;28(4):394–408. doi: 10.1016/j.devcel.2014.01.015 (PMC3992947; doi:10.1016/j.devcel.2014.01.015)
Supplement: Document S1. Supplemental Experimental Procedures, Figures S1–S4, and Table S1 [file mmc1.pdf]

Developmental Cell, Volume *28*

Supplemental Information

Enabled Negatively Regulates Diaphanous-Driven Actin Dynamics In Vitro and In Vivo

Colleen G. Bilancia, Jonathan D. Winkelman, Denis Tsygankov, Stephanie H. Nowotarski, Jennifer A. Sees, Kate Comber, Iwan Evans, Vinal Lakhani, Will Wood, Timothy C. Elston, David R. Kovar, and Mark Peifer

Figure S1.

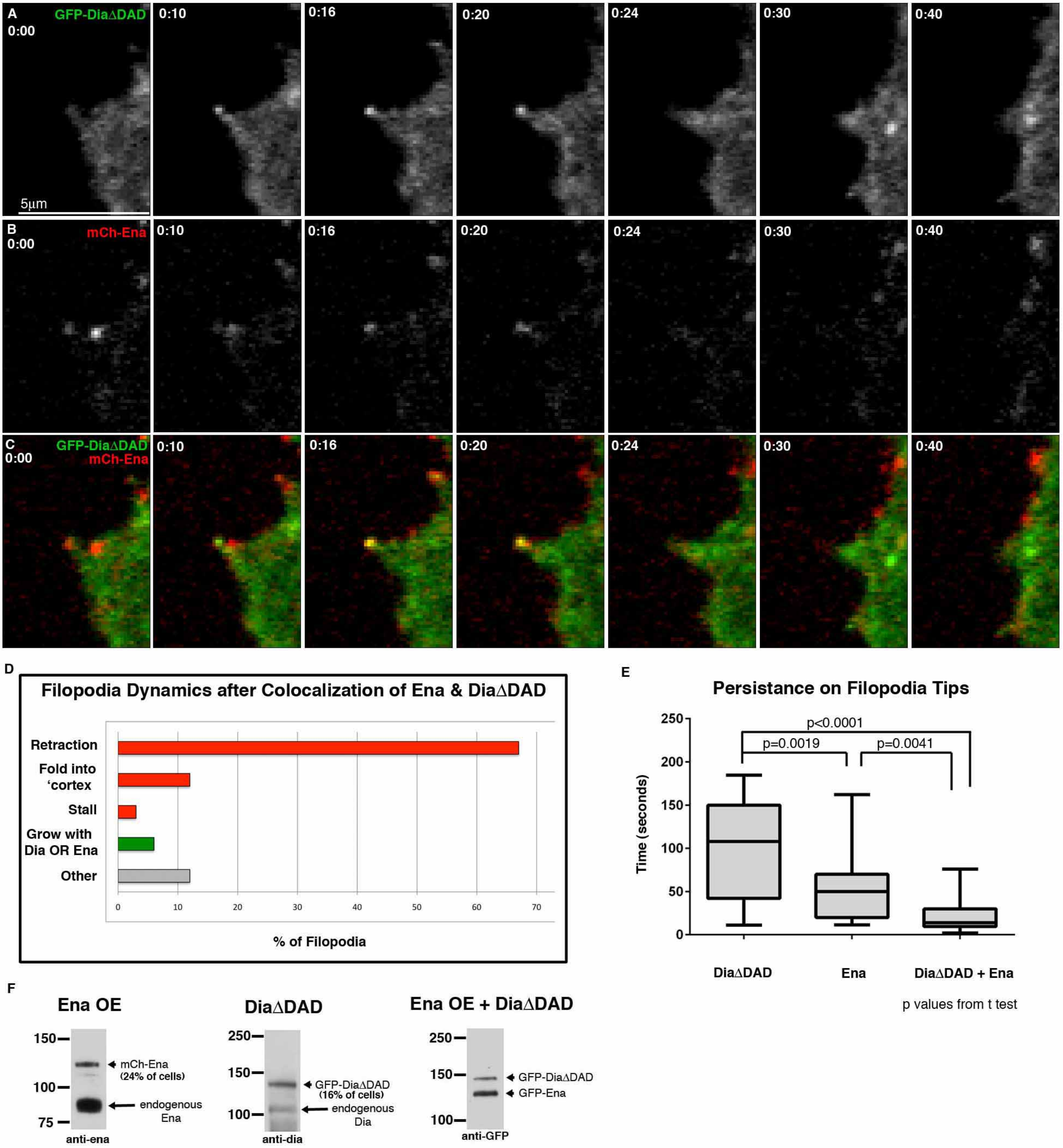
A
GFP-DiaΔDAD
0:00
0:10
0:16
0:20
0:24
0:30
0:40
5μm
B
mCh-Ena
C
GFP-DiaΔDAD
mCh-Ena
D
Filopodia Dynamics after Colocalization of Ena & DiaΔDAD
Retraction
Fold into 'cortex
Stall
Grow with Dia OR Ena
Other
0
10
20
30
40
50
60
70
% of Filopodia
E
Persistance on Filopodia Tips
250
200
150
100
50
0
Time (seconds)
p<0.0001
p=0.0019
p=0.0041
DiaΔDAD
Ena
DiaΔDAD + Ena
p values from t test
F
Ena OE
150
100
75
mCh-Ena
(24% of cells)
endogenous Ena
anti-ena
DiaΔDAD
250
150
100
GFP-DiaΔDAD
(16% of cells)
endogenous Dia
anti-dia
Ena OE + DiaΔDAD
250
150
100
GFP-DiaΔDAD
GFP-Ena
anti-GFP

Figure S2.

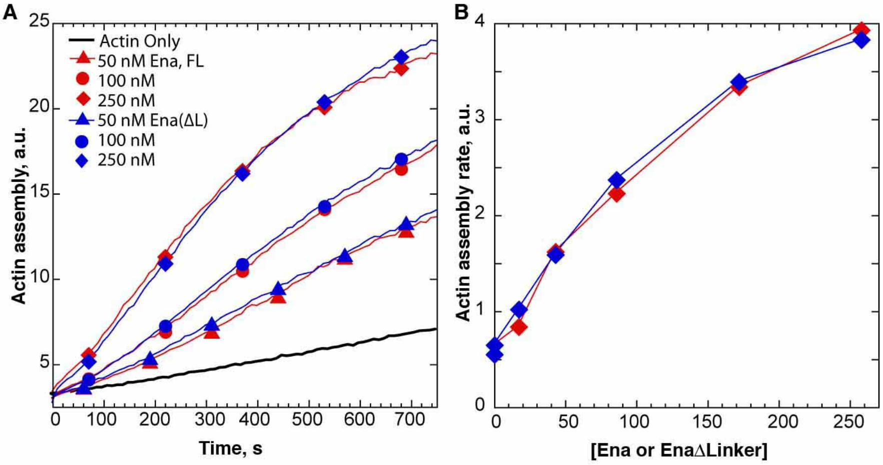
A
B
Actin Only
50 nM Ena, FL
100 nM
250 nM
50 nM Ena(ΔL)
100 nM
250 nM
Actin assembly, a.u.
Time, s
0
100
200
300
400
500
600
700
5
10
15
20
25
Actin assembly rate, a.u.
[Ena or EnaΔLinker]
0
50
100
150
200
250
1
2
3
4

Figure S3.

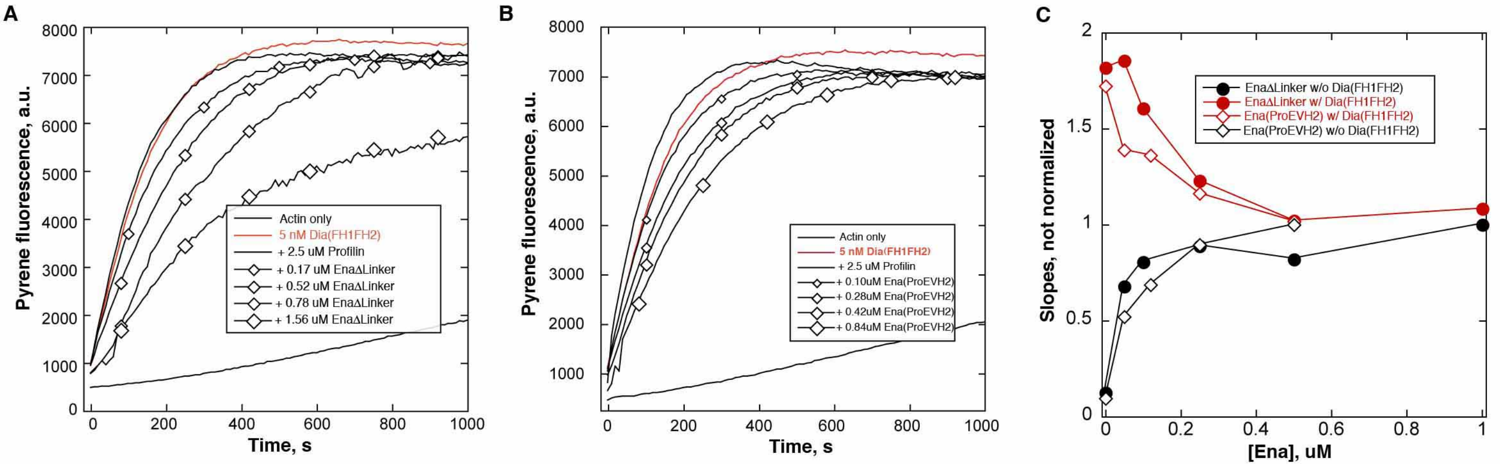
A
B
C
Pyrene fluorescence, a.u.
Time, s
Actin only
5 nM Dia(FH1FH2)
+ 2.5 uM Profilin
+ 0.17 uM EnaΔLinker
+ 0.52 uM EnaΔLinker
+ 0.78 uM EnaΔLinker
+ 1.56 uM EnaΔLinker
+ 0.10uM Ena(ProEVH2)
+ 0.28uM Ena(ProEVH2)
+ 0.42uM Ena(ProEVH2)
+ 0.84uM Ena(ProEVH2)
Slopes, not normalized
[Ena], uM
EnaΔLinker w/o Dia(FH1FH2)
EnaΔLinker w/ Dia(FH1FH2)
Ena(ProEVH2) w/ Dia(FH1FH2)
Ena(ProEVH2) w/o Dia(FH1FH2)

Figure S4.

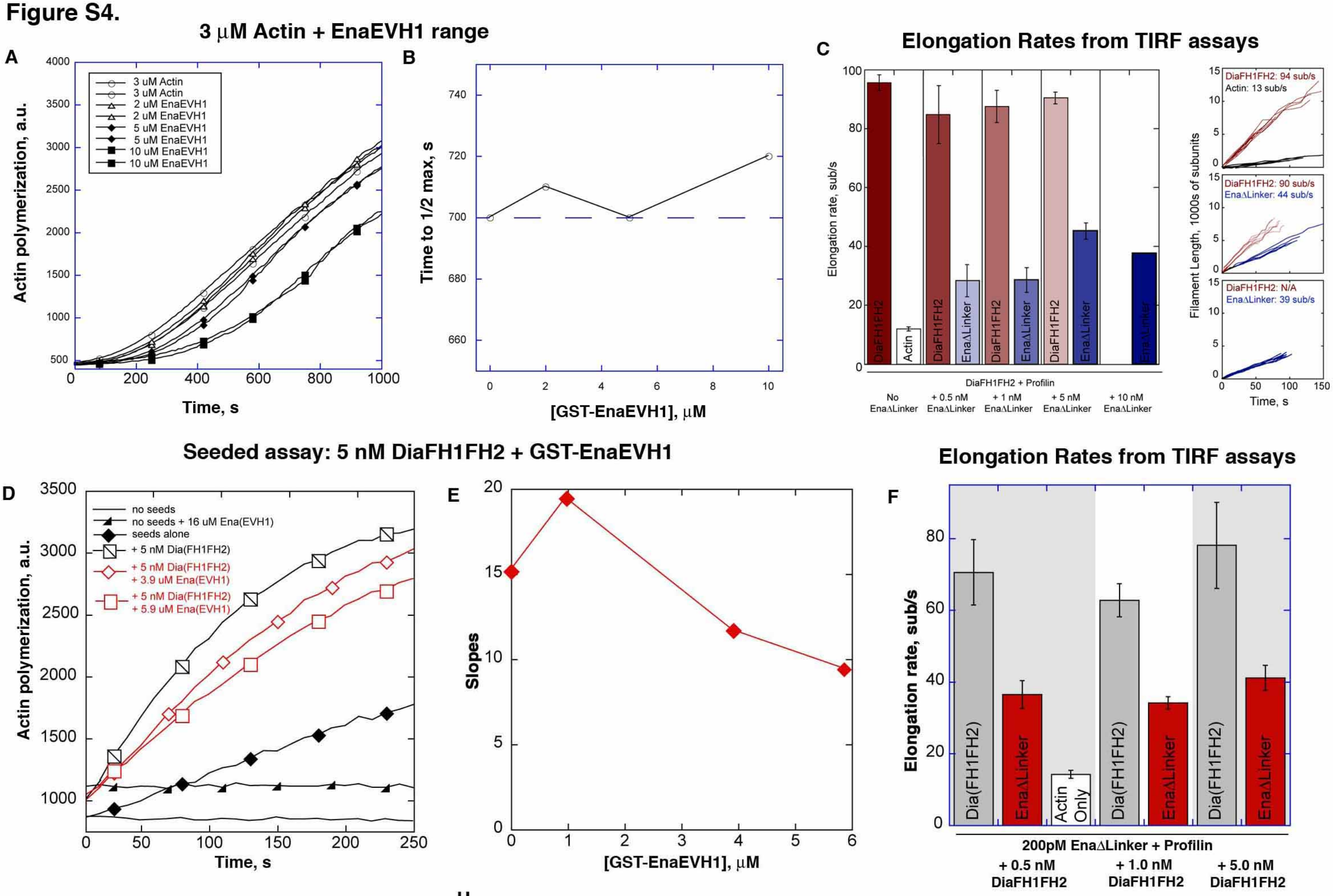

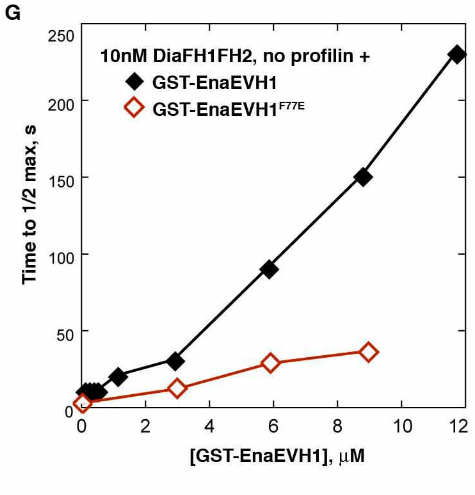

H

DiaFH1FH2 + GST-EnaEVH1 range, no profilin		
GST-EnaEVH1, uM	[Barbed Ends], nM	DiaFH1FH2 dimers/ nucleated filament
0	1.1	4.533
0	0.97	5.163
0.132	1.01	4.939
0.264	1	5
0.397	0.89	5.608
0.529	0.9	5.6
1.2	0.74	6.786
2.9	0.58	8.683
5.8	0.27	18.715
8.8	0.17	29.634
11.7	0.13	38.32

Supplemental Data

Supplemental Figures

Fig S1. Overexpression levels of tagged proteins and characterization of activated Dia and Ena colocalization in filopodia (related to Fig 2). A-C. Movie stills of cell expressing GFP-DiaΔDAD (A) and mCh-Ena (B,C=merge, Movie S3) showing filopodia retraction after colocalization of DiaΔDAD and Ena. Scale bar=5μm D. Quantification of filopodia dynamics after colocalization of DiaΔDAD and Ena. Retraction occurs in 67% of filopodia, 12% folded back into the cortex, and 3% stall. 6% of filopodia grew after colocalization but with only Ena or Dia on the tip. Finally 12% showed a complex morphology such as merging to adjacent filopodia. N=33 filopodia. E. Persistence on filopodia tips of DiaΔDAD, Ena, or their colocalization, measured in seconds. GFP-DiaΔDAD remains on filopodia tips for an average of 95s (31 filopodia from 7 cells). mCh-Ena remains on filopodia tips for an average of 56s (33 filopodia from 8 cells). GFP-DiaΔDAD and mCh-Ena remain on filopodia tips together for an average of 20s (14 filopodia from 9 cells). Box represents 75th to 25th percentile, whiskers show 90th and 10th percentile, line represents the mean, and p values calculated by t test. F. Western blot for Ena, Dia, or GFP to estimate overexpression levels of tagged proteins. Expression of tagged proteins (transfection efficiency = 24% for Ena and 16% for DiaΔDAD) compared to endogenous proteins shows Ena is ~3 fold overexpressed and Dia is ~30 fold overexpressed.

Figure S2. EnaΔLinker and full length (FL) Ena stimulate actin filament assembly similarly. **(related to Fig 3).** Spontaneous actin assembly assay with 2.5 μM Mg-ATP actin monomers (10% pyrene-actin). A. Time course of actin assembly in the absence (black curve) or presence of the indicated concentrations of EnaΔLinker (blue curve) or full-length Ena (red curve). B. Dependence of the linear phase of polymerization on the concentration of Ena (red curve) or EnaΔLinker (blue curve).

Figure S3. The assembly properties of Ena and Dia are not additive (related to Fig 5). Spontaneous assembly of 1.5 µM Mg-ATP actin monomers (10% pyrene-actin). A-B. Time course of actin assembly in the absence (black curve) or presence of 5 nM DiaFH1FH2 plus the indicated concentrations of EnaΔLinker (A) or EnaProEVH2 (B). C. Dependence of the linear phase of polymerization on the concentration of EnaΔLinker and EnaProEVH2 in the absence and presence of DiaFH1FH2. EnaΔLinker + 5 nM DiaFH1FH2 (red circles), EnaΔLinker alone (black circles). EnaProEVH2 + 5 nM DiaFH1FH2 (red diamonds) or EnaProEVH2 alone (black diamonds).

Figure S4. Role of Ena:Dia interactions on actin assembly (related to Fig 6). A,B. EnaEVH1 does not dramatically affect actin assembly. A. Raw curves from spontaneous actin assembly assays containing a range of GST-EnaEVH1 concentrations. Two samples were plotted for each condition. B. Time taken for each condition in A to reach ½ the maximal fluorescence. C. EnaΔLinker does not alter DiaFH1FH2 elongation rates. Left. Elongation rates from TIRF of actin filaments with SNAP-549-DiaFH1FH2 alone or with increasing concentrations of EnaΔLinker. Elongation rates fell into three populations: filaments with SNAP-549-DiaFH1FH2 on the barbed end (red), filaments growing at the Ena elongation rate (blue) or filaments growing at the actin-only rate (white). Error bars represent ±SEM, n=2. Right. Single filament traces from DiaFH1FH2 + Profilin (top), DiaFH1FH2 + Profilin + 5nm EnaΔLinker (middle), and DiaFH1FH2 + Profilin + 10 nM EnaΔLinker (bottom). D. GST-EnaEVH1 does not alter actin polymerization by DiaFH1FH2 in a seeded assembly assay. Raw curves of fluorescence vs. time for seeded assembly assays containing 5 nM DiaFH1FH2 and a range of GST-EnaEVH1 concentrations. E. Plots of the slopes of D during linear phase of actin assembly as a function of GST-EnaEVH1 concentration. F. Elongation rates from TIRF movies where reactions contained 200 pM SNAP-549-EnaΔLinker + Profilin and a range of DiaFH1FH2 concentrations. Error bars

are ±SD, grey columns are filaments displaying fast DiaFH1FH2 growth rates, red columns are rates measured from filaments with a SNAP-549-EnaΔLinker on the barbed end and the white column is the rate of actin alone internal controls. G. Profilin is not necessary for effects of EnaEVH1. Concentration of EnaEVH1 constructs vs. time to reach ½ steady-state fluorescence for 10nM DiaFH1FH2 in the absence of profilin (plot calculated from Fig 6A). H. Nucleation in the absence of profilin assessed by calculating the concentration of barbed ends nucleated by DiaFH1FH2 alone or with increasing concentrations of GST-EnaEVH1 and the average number of dimers required to nucleate a filament under the same conditions (see also Fig 6A,D).

Supplemental Movies

Movie S1. Dia and Ena induce protrusions distinct from one another and from those they induce when expressed together (stills of this movie are in Fig 2A-H'). Panel 1. Wildtype D16 cell expressing GFP-actin has actin-based filopodia and lamellipodia. Panel 2. D16 cell expressing mCh-Actin (white) and GFP-DiaΔDAD (not shown) shifts cell protrusions to long, stable filopodia. Panel 3. D16 cell expressing GFP-Actin (white) and mCh-Ena (not shown) drives dynamic filopodia that often emerge from fan-like protrusions. Panel 4. D16 cell expressing GFP-Actin and GFP-DiaΔDAD (white) plus mCh-Ena (not shown). Coexpression produces filopodia that are longer than wildtype but shorter than DiaΔDAD cells, and fan-like protrusions seen in Ena cells are absent. 100X magnification images were taken every 2s. Movie is displayed at 15 frames/second. Scale bar = 10μm. Time stamp is minutes:seconds.

Movie S2. Strong colocalization of DiaΔDAD and Ena at the cell cortex results in fewer filopodia (stills of this movie are in Fig 2I-K). Time-lapse confocal microscopy movie showing GFP-DiaΔDAD (A, green) and mCh-Ena (B, red) colocalization at the cell cortex (merge, C), which corresponds with a low number of filopodia. One filopodium forms at the end of the movie,

but only GFP-DiaΔDAD is present at the tip. 100X magnification images were taken every 2s. Movie is displayed at 15 frames/second. Scale bar = 1μm.

Movie S3. Colocalization of DiaΔDAD and Ena in filopodia often results in retraction (related to Fig 2, stills in Fig S1A-C). Time-lapse confocal microscopy movie showing GFP-DiaΔDAD (A, green) and mCh-Ena (B, red) in a filopodium. Colocalization (merge, C) is quickly followed by retraction of the filopodium. 100X magnification images were taken every 2s. Movie is displayed at 15 frames/second. Scale bar = 1μm.

Movie S4. Spontaneous actin assembly alone, in the presence of profilin and EnaΔLinker, or profilin and DiaFH1FH2 (stills of this movie in Fig 3). Arrowheads mark filament barbed ends when unoccupied (open), or with EnaΔLinker or Dia (red). Top: TIRF movie of 1.5 µM actin (15% Oregon green-labeled; green). Control for DiaFH1FH2 and EnaΔLinker. Middle: EnaΔLinker increases actin assembly. TIRF movie of 1.5 µM actin (15% Oregon green labeled; green), Drosophila profilin (Chickadee), and quantum dot-labeled biotin-SNAP-EnaΔLinker (red). Bottom: DiaFH1FH2 increases actin assembly. TIRF movie of 1.5 µM actin (15% Oregon green-labeled; green), Drosophila profilin, and quantum dot-labeled biotin-SNAP-DiaFH1FH2 (red). Movie is displayed at 15 frames/second. Time is minutes:seconds.

Movie S5. Spontaneous actin assembly of DiaFH1FH2, EnaEVH1, or both in the absence of profilin. (Stills of this movie are in Fig 6). Top: Actin assembly is largely unaffected in the presence of EnaEVH1 although actin puncta form. TIRF movie of 1.5 µM actin (15% Oregon green-labeled; green) + 5 µM GST-EnaEVH1 (unlabeled) in the absence of profilin. Movie shows actin puncta formation, although this has little effect on actin assembly in pyrene assays (Fig S4A,B). Circle marks filament pointed end; arrowhead marks the filament barbed end.

Middle: DiaFH1FH2 actin assembly in the absence of profilin. TIRF movie of 1.5 μM actin (15% Oregon green-labeled; green) + 1 nM SNAP-549-DiaFH1FH2 (red) in the absence of profilin. Circle marks filament pointed end; red arrowhead marks the filament barbed end with DiaFH1FH2. Bottom: EnaEVH1 inhibits actin assembly by DiaFH1FH2. TIRF movie of 1.5 μM actin (15% Oregon green-labeled; green) + 1 nM SNAP-549-Dia-FH1FH2 (red) + 5 μM GST-EnaEVH1 (unlabeled) in the absence of profilin. Movie shows green spots of actin accumulating in the presence of GST-EnaEVH1. Many of these colocalize with DiaFH1FH2 (red). White arrow marks EVH1/actin puncta. Eventually, a DiaFH1FH2 elongated filament emerges from the actin-DiaFH1FH2 puncta (red arrowheads). Movie is displayed at 15 frames/second. Time is minutes:seconds.

Movie S6. Wildtype *Drosophila* embryonic dorsal closure (stills of this movie are in Fig 7C). Wildtype dorsal closure in an embryo expressing GFP-Actin using *engrailed*-GAL4. Note endogenous Ena present. Control for Movie S7. 100x magnification images were collected every 5s. Movie is displayed at 15 frames/second. Scale bar = 10μm. Time is hours:minutes:seconds.

Movie S7. Dia-driven protrusions are more dynamic in areas of high Ena accumulation during *Drosophila* embryonic dorsal closure (stills of this movie are in Fig 7D). Dorsal closure in an embryo expressing both GFP-Actin and GFP-DiaΔDAD using *engrailed*-GAL4. Endogenous Ena is enriched at the leading edge and at tricellular junctions relative to lateral cell borders. 100x magnification images were collected every 5s. Movie is displayed at 15 frames/second. Scale bar = 10μm. Time is hours:minutes:seconds.

Supplemental Tables

Supplemental Table 1. Yeast two-hybrid interactions between Dia and Ena.					
	**Dia**	**DiaΔDAD**	**DiaGBD**	**DiaARR-DD**	**Dia GBD-ARR-DD**
**AD**	28.6 ±11.6	42.2 ±6.51	103 ±27.9	197 ±2.45	126 ±15.5
**Ena**	11.3 ±1.83	17.7 ±0.924	61.5 ±23.5	38.9 ±6.71	35.6 ±19.4
**EnaEVH1**	15.1 ±2.65	42.6 ±10.9	51.3 ±27.1	147 ±6.71	32.8 ±10.2
**EnaLinkerPro**	16.6 ±6.27	24.6 ±13.6	41.4 ±11.2	29.1 ±3.76	61.5 ±29.2
**EnaEVH2**	8.59 ±3.43	18.3 ±3.10	40.5 ±23.0	69.6 ±7.84	16.7 ±8.53
	**DiaFH1**	**DiaFH2**	**DiaFH1FH2**	**DiaFH1FH2DAD**	**DiaDAD**
**AD**	41.2 ±22.2	264 ±61.1	138 ±59.2	29.5 ±16.7	28.1 ±12.6
**Ena**	12.6 ±3.73	74.1 ±45.6	91.1 ±15.5	13.3 ±7.88	10.5 ±7.50
**EnaEVH1**	**903 ±797**	186 ±154	872 ±1620	9.26 ±0.725	16.5 ±7.16
**EnaLinkerPro**	10.5 ±3.66	2520 ±2780	4.89 ±1.82	24.9 ±19.8	16.4 ±5.49
**EnaEVH2**	22.7 ±9.68	87 ±28.0	113 ±33.9	7.23 ±1.06	5.25 ±0.872
	**Ena**	**EnaEVH1**	**EnaLinkerPro**	**EnaEVH2**	
**AD**	22.1 ±7.26	165 ±102	52.1 ±11.4	39.5 ±24.7	
**Dia**	13 ±5.54	**708 ±749**	29.5 ±7.32	18.6 ±4.22	
**DiaΔDAD**	14.4 ±9.22	142 ±195	35.7 ±27.2	8.97 ±1.22	
**DiaGBD**	10.7 ±1.29	107 ±9.8	19.9 ±8.54	11.5 ±0.589	
**DiaARR-DD**	11.3 ±7.36	40.8 ±8.58	15.7 ±15.9	34.3 ±21.2	
**DiaGBD-ARR-DD**	8.33 ±5.32	20.8 ±9.83	12.1 ±7.41	14.9 ±8.51	
**DiaFH1**	31.1 ±4.59	93.9 ±/23.2	126 ±160	28.8 ±10.9	
**DiaFH2**	7.07 ±0.483	61.2 ±25.0	44.3 ±19.2	55.4 ±12.2	
**DiaFH1FH2**	**44.8 ±14.7**	**804 ±287**	53.1 ±43.9	10.3 ±3.95	
**DiaFH1FH2DAD**	15.9 ±2.15	**382 ±220**	**85.5 ±32.0**	27.8 ±1.39	
**DiaDAD**	28.7 ±9.79	191 ±120	104 ±40.8	29.6 ±6.83	
**FP4**		**12400 ±680**			

Table S1. Yeast two-hybrid interactions between Ena and Dia (related to Fig 4). Results from liquid βgalactosidase assays shown as mean Miller units ± SD. Bait constructs are listed horizontally and prey constructs are listed vertically. The activation domain (AD) was used to measure background auto-activation by each bait construct. Results in bold represent constructs that increase the mean Miller units above that of the bait+AD with a p-value < 0.05 based on the Mann-Whitney U test.

Supplemental Experimental Procedures

Fly stocks	Source
UAS-GFP-Actin	P. Martin, University of Bristol, UK
UAS-GFP-DiaΔDAD	Homem and Peifer, 2009
UAS-GFP-Ena	Gates et al., 2007
snGAL4, UAS-LifeActGFP	B. Stramer, King's College London, UK
*srp*GAL4	Brückner et al., 2004
*crq*GAL4	Stramer et al., 2005
UAS-mCh-Moesin	Millard and Martin, 2008
*en*GAL4	Bloomington *Drosophila* Stock Center
UAS-redstinger	Bloomington *Drosophila* Stock Center
UAS-GFP	Bloomington *Drosophila* Stock Center

Antibodies/Probes	Dilution	Source
anti-Dia	1:5000	S. Wasserman, UCSD, CA,
anti-Ena	1:100 (WB)<br>1:500 (IF)	Developmental Studies Hybridoma Bank, University of Iowa, Iowa City, IA
anti-GFP (JL-8)	1:5000	Clontech, Mountain View, CA
TRITC-phalloidin	1:1000	Simga-Aldrich, St. Louis, MO
Alexa-488, 568, and 647	1:500	Life Technologies, Carlsbad, CA
HRP-conjugated goat anti-mouse or anti-rabbit	1:50,000	Pierce Antibodies, ThermoFisher Scientific, Waltham, MA
SuperSignal West Dura Chemiluminescent Substrate		ThermoFisher Scientific, Waltham, MA

Cell Culture

Drosophila D16C3 cells were cultured in Schneider's Media supplemented with 10% fetal bovine serum and 10µg/mL recombinant human insulin (Gibco, Life Technologies, NY, USA). For live imaging, FugeneHD (Promega, WI, USA) was used to transfect cells. Cells were plated on poly-d-lysine coated glass bottom dishes (MatTek Corporation, MA, USA) and imaged 48-72 hours later. Transfection efficiency ranged from 10-25% and expression levels were variable. Cells with mid-range expression (by eye) were used for all experiments. Cells were imaged in Schneider's media at room temperature. Images were acquired every 2 seconds for a minimum of 2-6 minutes using the 100X 1.4NA objective on a Wallac Ultraview Confocal Imaging System (PerkinElmer, MA, USA). Brightness and contrast were adjusted using ImageJ (NIH, MD, USA). Quantification was carried out $\geq$ 60 frames from 10-35 cells using our computational method (number and length) or manually (lifetime and persistence) where filopodia were defined as at least 1μm long and less than 0.77μm wide.

Immunohistochemistry and western blots

D16C3 cells were plated on glass cover slips and fixed with 32% paraformaldehyde solution (Electron Microscopy Sciences, PA) diluted to 10% in PBS. Cells were permeabilized with 0.1% TritonX-100, blocked with 5% normal goat serum, and stained for Ena, Dia, and actin. Samples were mounted in Aqua/PolyMount (Polysciences, Inc., PA, USA) and imaged as above. For overexpression western blots, D16 cells were transfected and samples were plated, fixed, and stained for phalloidin as described above. The remaining sample was lysed and run on an SDS-PAGE gel, transferred to nitrocellulose, and blotted for Ena, Dia, or GFP, HRP-conjugated goat anti-rabbit or mouse, and SuperSignal West Dura Chemiluminescent Substrate. For measuring approximate expression levels of tagged proteins, relative band intensities for tagged versus endogenous protein were measured for at least 2 lanes in $\geq$3 different exposures using ImageJ (NIH, USA). 120 stained cells were counted for each genotype to measure transfection

efficiency (24% for Ena and 16% for DiaΔDAD), and calculated the level of expression (~3 fold over endogenous Ena; ~30 fold over endogenous Dia).

CellGeo Computational Method

To automate the identification and quantification of filopodia we developed the computational platform *CellGeo* (Tsygankov et al., 2014). *CellGeo* is a MATLAB application with an easy to use Graphical User Interface (GUI).

Method: The key component of our computational tools for cell shape analysis is the representation of 2 dimensional cell geometries as tree-graphs (specifically, as Voronoi graphs built inside closed piecewise linear curves). Because we do not use any of the typical pruning or edge reduction algorithms, the Voronoi graph provides a distance measure for every boundary point to a unique internal point (the graph root) along a well-defined path inside the cell. The benefit of this distance measure (boundary profile) is that it allows the identification of filopodia regardless of the complexity of cell geometry.

By construction, each point on the path from a filopodia tip to the graph root is the center of an inscribed circle within the cell boundary and, thus, the radii of these circles provide a natural measure of protrusion half-width. A critical radius length is used to define the base of filopodia. The length of the path from the base to the tip of the filopodia defines the filopodia length. A distance measure based on full “filopodia skeletons” (the paths from base to tip), allows *CellGeo* to track filopodia over time and measure dynamic characteristics, such as lifetime and protrusion and retraction rates.

Software: *CellGeo*’s pipeline consists of five modules. The segmentation module, *MovThresh*, allows automatic extraction and visualization of cell boundaries. *MovThresh* also allows interactive adjustment of time-dependent threshold values to ensure proper identification of thin filopodia. The second module, *BisectoGraph*, constructs the tree-graphs and cell

boundary profiles. This module allows batch processing. The remaining three modules, *FiloTrack*, *ProActive*, and *ConeTrack*, use the results of *BisectoGraph* to quantify three different types of protrusions: filopodia, broad protrusions and neuronal growth cones. *FiloTrack* allows interactive specification of critical parameters that define protrusions of interest and customization of the tracking algorithm. *ProActive* (not used in this study) visualizes protruding and retracting parts of the cell and quantifies broad motion of the cell body. *ConeTrack* (not used in this study) identifies neuronal growth cones for the analysis of their dynamic properties, such as shape change, protrusion and retraction rates.

Protein Expression and Purification

Recombinant Diaphanous and Enabled proteins were purified by expressing in E. coli strain BL21-Codon Plus (DE3)-RP (Stratagene, CA, USA) with 0.5 mM IPTG for 16 h at 16 °C. Harvested cells were resuspended in extraction buffer (50mM NaH2 PO4, pH 8.0, 500mM NaCl, 10% glycerol, 10 mM imidazole, 10 mM β-mercaptoethanol) supplemented with protease inhibitors and homogenized in an Emulsi-Flex-C3 (Avestin, Canada). The homogenate was clarified at 30,000 (15 min) and 50,000 x g (30 min), and the extract was incubated with Talon Metal Affinity Resin (Clontech, CA, USA) for 1 h at 4 °C and then loaded onto a disposable column. After a 50-ml wash with extraction buffer, proteins were eluted with Talon elution buffer (50 mM NaH2 PO4, pH 8.0, 500 mM NaCl, 10% glycerol, 10 mM β-mercaptoethanol, 250mM imidazole). Ena constructs were gel purified on S200 10/300 GL column (GE Healthcare, Sweden) and then eluted as stable tetramers. Gel filtration did not affect protein activity, so this step was omitted in some assays. Dia constructs were dialyzed against formin buffer (20mM Hepes, pH 7.4, 1 mM EDTA, 200 mM KCl, 0.01% NaN3, and 1 mM DTT) and flash-frozen in liquid nitrogen and stored at -80 °C. Full-length Ena lost activity upon freeze/thaw, and slowly lost activity after purification so it was utilized within 1 week. For expression of SNAP-tagged

proteins, Dia and Ena constructs were sub-cloned from pET21a vectors into the SNAP-tag-T7-2-vector (NEB, MA, USA) at the PacI/NotI sites. A flexible Linker (GGSGGS) was introduced between the SNAP tag and Ena or Dia start codon. SNAP-tagged proteins were labeled according to manufacturer's protocols and then incubated overnight at 4 °C. Excess label was removed either by dialysis into Formin buffer without EDTA or gel filtration on a S200 column.

Total Internal Reflection Fluorescence Microscopy

Microscope slides and cover slips (#1.5, Fisher Scientific, PA, USA) were washed with acetone (30 min), 95% ethanol (10 min) and sonicated with 1M NaOH (10 min), 1M HCl (10 min) and Helmanex detergent (2 hr). Glass was then washed with deionized water, dried, and incubated for 18 hr with 1mg/ml mPeg-Silane (MW=5000) dissolved in 95% ethanol pH 2. Parallel strips of double-sided tape were placed on coverslips to create multiple flow chambers per cover glass.

TIRF microscopy images were collected at 2-4s intervals with an iXon EMCCD camera (Andor Technology, UK) using an Olympus IX-71 microscope fit with through-the-objective TIRF illumination. Mg-ATP-actin (15% Oregon green–labeled) was mixed with 2X TIRF buffer (10mM Imidazole, pH 7.0, 50mM KCl, 1mM MgCl2, 1mM EGTA, 50mM DTT, 0.2mM ATP, 50μM CaCl2, 15mM glucose, 20μg/ml catalase, 100μg/ml glucose oxidase and 0.5% (w/v) methylcellulose (viscosity 400 centipoise) and Ena or Dia constructs (with or without 3.0μM profilin), and transferred to a flow cell for imaging at 23 °C. For two-color TIRF, we cyclically imaged Oregon-green actin (1 frame, 488nm excitation for 50ms) and SNAP or quantum dot conjugates (1 frame, 561nm excitation for 50ms). For quantum dot experiments, biotinylated SNAP-tagged proteins were incubated with streptavidin-conjugated quantum dots (QD-625, Invitrogen, CA, USA). To cluster Ena onto QD-625, the dots were incubated with a 10:1 molar excess of Ena. Both 488 and 561 lasers excite QD-625 and emission can be detected in both these channels.

To calculate residence times, association of Ena or Dia with the barbed end was tracked manually. An association event had several criteria: 1) the filament had to be actively

elongating, 2) the association had to last for two or more frames and 3) due to an increase in filament bundling in the presence of GST-EnaEVH1 we did not include data from Dia-associated barbed ends that became incorporated into bundles. For SNAP-549-DiaFH1FH2 constructs, the filaments often elongated out of the field of view. We therefore used a 300s window. Filament elongation rates were calculated by measuring filament lengths over time with ImageJ (NIH, USA). All curve fitting data and plots were made in KaleidaGraph (Synergy Software, PA, USA).

Fluorescence Spectroscopy

Actin assembly was measured from the fluorescence of pyrene-actin with Safire^2 fluorescent plate reader (Tecan, CA, USA). Spontaneous assembly has been described in detail previously (Neidt et al., 2008). Briefly, the assembly of 10% pyrene-labeled Mg-ATP-actin monomers was initiated by the addition of 50mM KCl, 1mM $MgCl_2$, 1mM EGTA, 10mM imidazole, pH 7.0, and other proteins to be assayed (Ena, Dia, etc.). Final protein concentrations are indicated in the figure legends. Calculation of spontaneous polymerization rates has been described.

Nucleation Data

Data taken from 1.5 uM actin, 10% pyrene-labeled. Equation used: [barbed ends] = bulk elongation rate/(k+[actin monomers]), where k+ = 16.7 uM-^{1} s-^{1}. We assumed that the elongation rate did not change as a function of GST-EnaEVH1, and that all of the nucleation happens at the beginning of the reaction. We also assumed that all barbed ends were bound to DiaFH1FH2 and elongating at the DiaFH1FH2 rate. The plateau of the pyrene curves were set = 1.4 μM actin. The slope (bulk elongation rate) of the curves was calculated at 33% assembly, when the reaction was in linear phase. To calculate the average number of dimers required to nucleate a filament, the concentration of DiaFH1FH2 (10 nM/5 nM dimer) was divided by the number of barbed ends.

Yeast Two-hybrid

Yeast two-hybrid analysis was performed using the LexA system and strain EGY48 (Finley and Brent, 1996) containing the pSH18-34 LacZ reporter plasmid. pEG202 plasmid was used to generate bait constructs fused to the DNA Binding Domain and pJG4 was used for prey constructs fused to the Activation Domain. Bait and prey of Ena and Dia were tested pair wise for their ability to bind. Bait and prey were co-transformed and grown on 10% glucose –His -Trp –Ura selective media. Individual colonies were grown overnight in 20% galactose/20% raffinose –His –Trp –Ura liquid media to induce LacZ expression. LacZ was measured by liquid βgalactosidase activity assays as described (Pai et al, 1996). Each bait construct was co-transformed with the empty activation domain plasmid as a control. At least three assays were performed for each bait-prey pair, with two βgalactosidase reactions per assay. Results were compared statistically using the Mann-Whitney U test.

GST Pull-down

Ena and Dia domain constructs were N-terminally tagged with GST (pGEX plasmid) or MBP (pET21a plasmid) and C-terminally with 6x His. Constructs were expressed in BL-21 (DE) Gold or BL21 (DE)-RP cells (Stratagene, CA, USA). Overnight cultures were grown at 37°C and used to seed large cultures, which were induced by 0.5mM IPTG and grown overnight at 18°C. Bacteria were pelleted, washed in PBS, and lysed by sonication, followed by 30-minute spin and collection of the lysates. Bacterial lysates were mixed and incubated with 50μL bed volume of Glutathione-Sepharose-4B (GE Healthcare, Sweden) for 2 hr at 4°C. Beads were pelleted and supernatant samples were collected. Remaining supernatant was removed and beads were washed three times with PBS. Protein sample buffer was added to all samples, they were boiled for 5 min, and spun down for 5 min. Samples were run on SDS-PAGE gels and stained with Coomassie or western blotted. Coomassie staining was performed for 20 min at room

temperature on a shaker, followed by overnight destaining. Westerns were performed using Dia antibody, HRP-conjugated goat anti-rabbit, and SuperSignal West Dura Chemiluminescent Substrate.

***Drosophila* Image Acquisition and Quantification:** For dorsal closure live imaging, embryos were dechorionated in 50% bleach, mounted in halocarbon oil (series 700; Halocarbon Products, River Edge, NJ) between a cover slip and gas permeable membrane (Petriperm; Sartorius, Edgewood, NJ). Single plane images were acquired at 5s intervals using a 100X 1.4 NA Plan Apo VC Nikon objective on an inverted TE2000-E microscope (Nikon, Tokyo, Japan) with a Visitech VTHawk confocal system (VisiTech, Sunderland, UK). Images were acquired on an Orca R2 CCD camera (Hammamatsu Photonics K.K., Japan). Image J (NIH, MD, USA) was used for quantification of filopodia persistence and maximum length, and were defined as any thin protrusion (width < 1.15 μM, length > 1.15 μM) extending beyond the lamellipodium or leading edge. Quantification was performed on 1 or more pairs of contralateral stripes (as the leading edge moved from 30-15 μM apart) per embryo, with 3 or more embryos analyzed per genotype. Statistics were performed using a one-tailed t Test.

Live imaging of inflammatory migration was performed on GFP-expressing hemocytes in stage 15 embryos prepared as previously described (Wood and Jacinto, 2005) and imaged on a spinning disc confocal microscope (Ultraview, PerkinElmer). Images were captured for 1h post-wounding at 1min intervals. Hemocytes were tracked with ImageJ (NIH). Hemocyte morphology, filopodia and actin bundles were quantified from still images of LifeAct-expressing hemocytes.

Supplemental References

Brückner, K., Kockel, L., Duchek, P., Luque, C.M., RØrth, P., Perrimon, N. (2004). The PDGF/VEGF receptor controls blood survival in Drosophila. Dev. Cell. *7*, 73-84.

Finley, R.L. and Brent, R. (1996). Interaction trap cloning in yeast. In Gene Probes: A Practical Approach (Oxford: Oxford University Press).

Gates, J., Mahaffey, J.P., Rogers, S.L., Emerson, M., Rogers, E.M., Sottile, S.L., Van Vactor, D., Gertler, F.B., and Peifer, M. (2007). Enabled plays key roles in embryonic epithelial morphogenesis in Drosophila. Development *134*, 2027-2039.

Millard, T.H. and Martin, P. (2008). Dynamic analysis of filopodial interaction during the zippering phase of Drosophila dorsal closure. Development *135*, 621-626.

Neidt, E.M., Skau, C.T., and Kovar, D.R. (2008). The cytokinesis formins from the nematode worm and fission yeast differentially mediate actin filament assembly. J Biol Chem. *283*, 23872-23883.

Pai, L.M., Kirkpatrick, C., Blanton, J., Oda, H., Takeichi, M., and Peifer, M. (1996). Drosophila alpha-catenin and E-cadherin bind to distinct regions of Drosophila Armadillo. J Biol Chem. *271*, 32411-32420.

Stramer, B., Wood, W., Galko, M.J., Redd, M.J., Jacinto, A., Parkhurst, S.M., and Martin, P. (2005). Live imaging of wound inflammation in Drosophila embryos reveals key roles for small GTPases during in vivo cell migration. J Cell Biol. *168*, 567-573.

Tsygankov, D., Bilancia, C.G., Vitriol, E.A., Hahn, K.M., Peifer, M., and Elston, T.C. (2014). CellGeo: a computational platform for the analysis of shape changes in cells with complex geometries. J. Cell Biol. Published online February 3, 2014. 10.1083/jcb.201306067

Wood, W. and Jacinto, A. (2005). Imaging cell movement during dorsal closure in Drosophila embryos. Methods Mol Biol *294*, 203-210.
