# Supplementary figures and images for: Enabled Negatively Regulates Diaphanous-Driven Actin Dynamics In Vitro and In Vivo
Source: Dev Cell. 2014 Feb 24;28(4):394–408. doi: 10.1016/j.devcel.2014.01.015 (PMC3992947; doi:10.1016/j.devcel.2014.01.015)

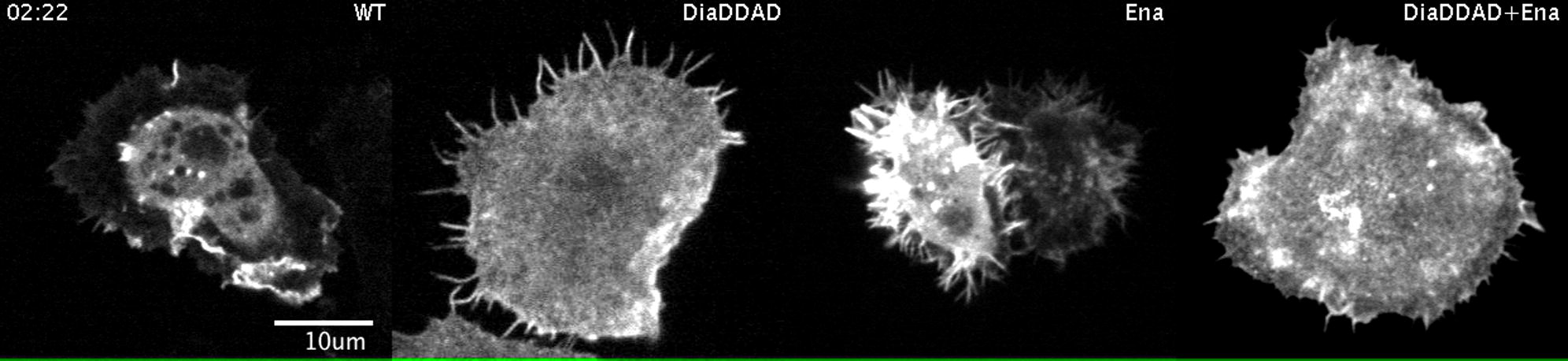

Supplement: Movie S1, Related to Figure 2. Dia and Ena Induce Protrusions Distinct from One Another and from Those They Induce When Expressed Together — Panel 1: Wild-type D16 cell expressing GFP-Actin has actin-based filopodia and lamellipodia. Panel 2: D16 cell expressing mCh-Actin (white) and GFP-DiaΔDAD (not shown) shifts cell protrusions to long, stable filopodia. Panel 3: D16 cell expressing GFP-Actin (white) and mCh-Ena (not shown) drives dynamic filopodia that often emerge from fan-like protrusions. Panel 4: D16 cell expressing GFP-Actin and GFP-DiaΔDAD (white) plus mCh-Ena (not shown). Coexpression produces filopodia that are longer than wild-type but shorter than DiaΔDAD cells, and fan-like protrusions seen in Ena cells are absent. Images (100× magnification) were taken every 2 s. Movie is displayed at 15 frames/s. Scale bar represents 10 μm. Time stamp is min:s. [file mmc2.jpg]

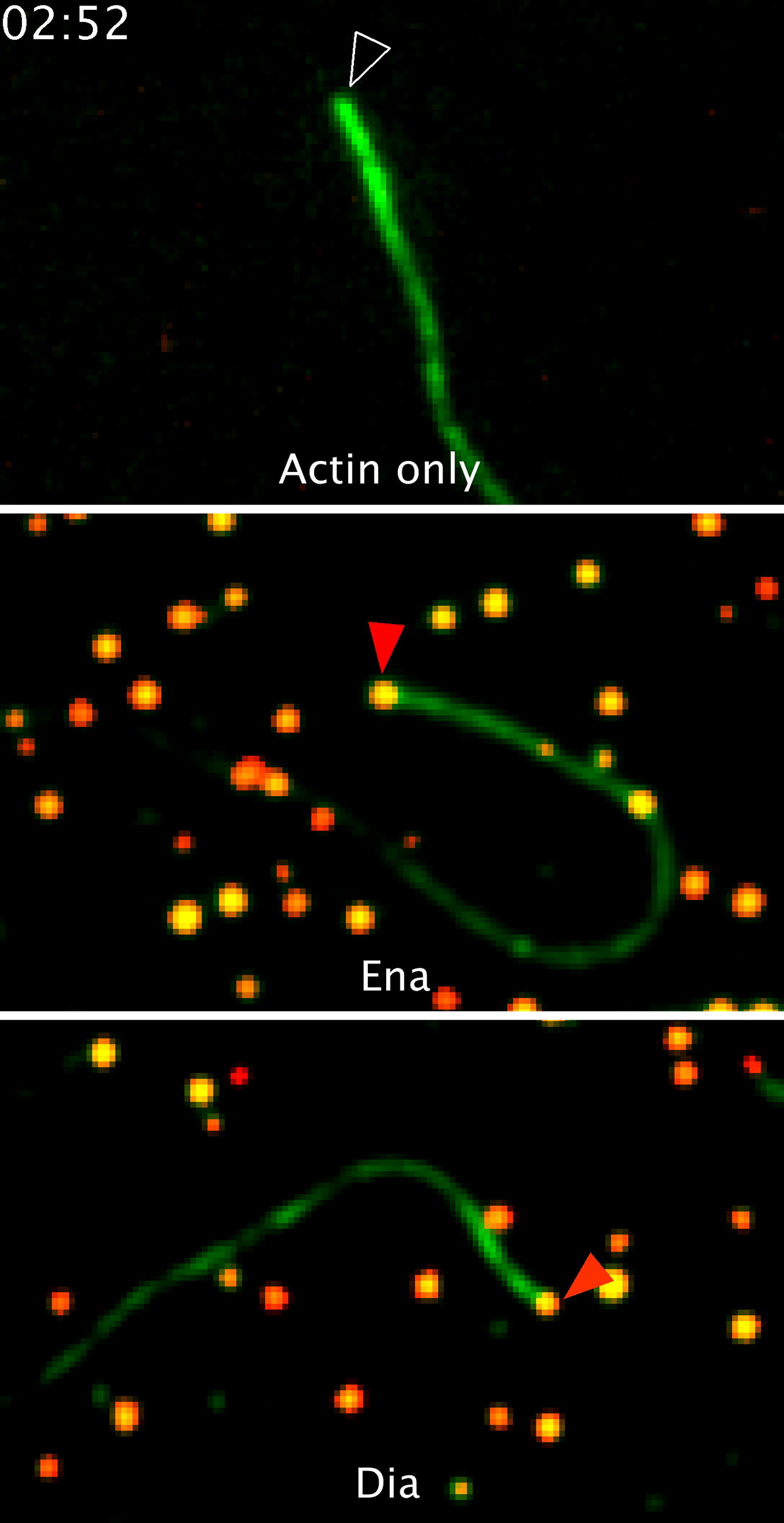

Supplement: Movie S2, Related to Figure 3. Spontaneous Actin Assembly Alone, in the Presence of Profilin and EnaΔLinker, or in the Presence of Profilin and DiaFH1FH2 — Arrowheads mark filament barbed ends when unoccupied (open) or when occupied with EnaΔLinker or Dia (red). Top: TIRF movie of 1.5 μM actin (15% Oregon-green-labeled; green). Control for DiaFH1FH2 and EnaΔLinker. Middle: EnaΔLinker increases actin assembly. TIRF movie of 1.5 μM actin (15% Oregon-green-labeled; green), Drosophila profilin (Chickadee), and quantum dot-labeled biotin-SNAP-EnaΔLinker (red). Bottom: DiaFH1FH2 increases actin assembly. TIRF movie of 1.5 μM actin (15% Oregon-green-labeled; green), Drosophila profilin, and quantum dot-labeled biotin-SNAP-DiaFH1FH2 (red). Movie is displayed at 15 frames/s. Time is min:s. [file mmc3.jpg]

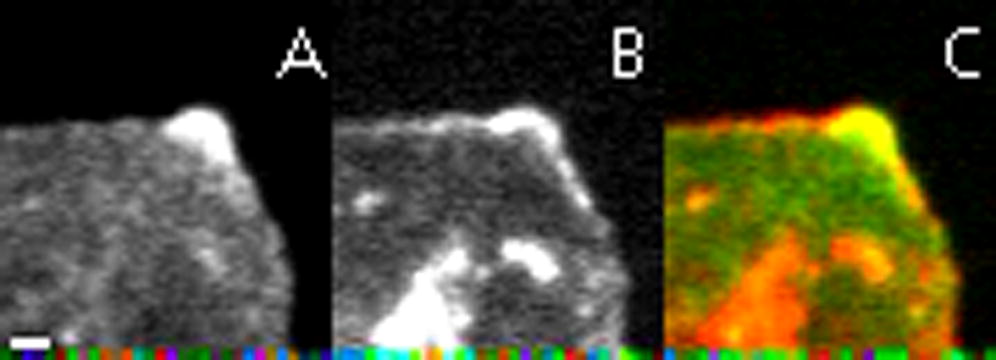

Supplement: Movie S3, Related to Figure 2. Strong Colocalization of DiaΔDAD and Ena at the Cell Cortex Results in Fewer Filopodia — Time-lapse confocal microscopy movie showing GFP-DiaΔDAD (A, green) and mCh-Ena (B, red) colocalization at the cell cortex (merge, C), which corresponds with a low number of filopodia. One filopodium forms at the end of the movie, but only GFP-DiaΔDAD is present at the tip. Images (100× magnification) were taken every 2 s. Movie is displayed at 15 frames/s. Scale bar represents 1 μm. [file mmc4.jpg]

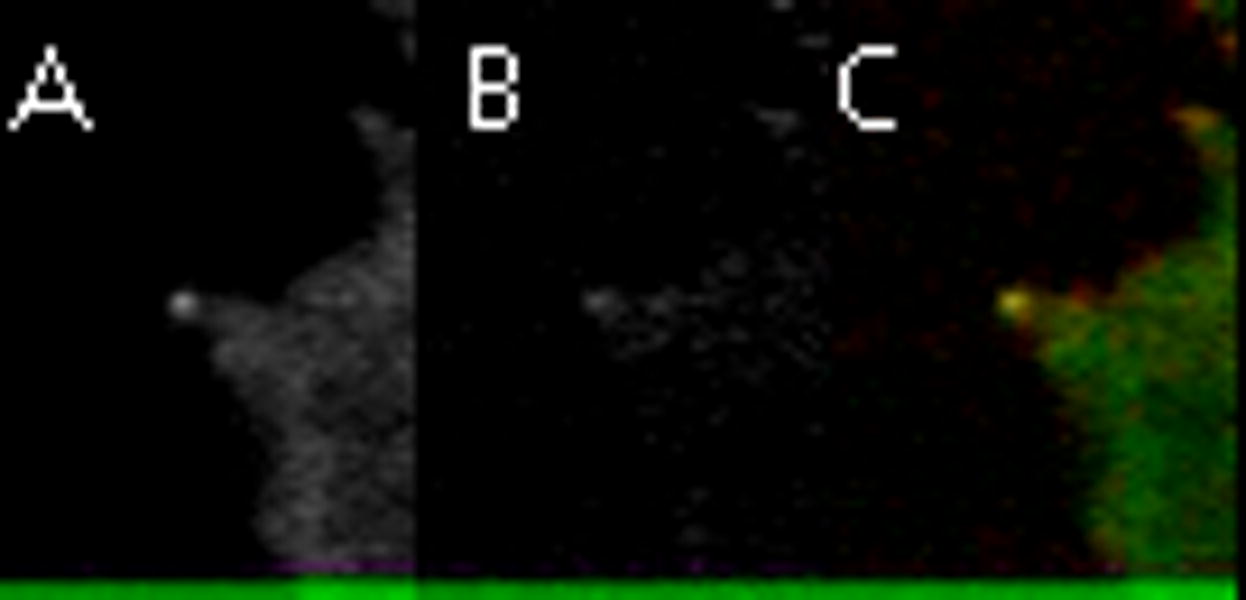

Supplement: Movie S4, Related to Figure 2. Colocalization of DiaΔDAD and Ena in Filopodia Often Results in Retraction — Time-lapse confocal microscopy movie showing GFPDiaΔDAD (A, green) and mCh-Ena (B, red) in a filopodium. Colocalization (merge, C) is quickly followed by retraction of the filopodium. Images (100× magnification) were taken every 2 s. Movie is displayed at 15 frames/s. Scale bar represents 1 μm. [file mmc5.jpg]

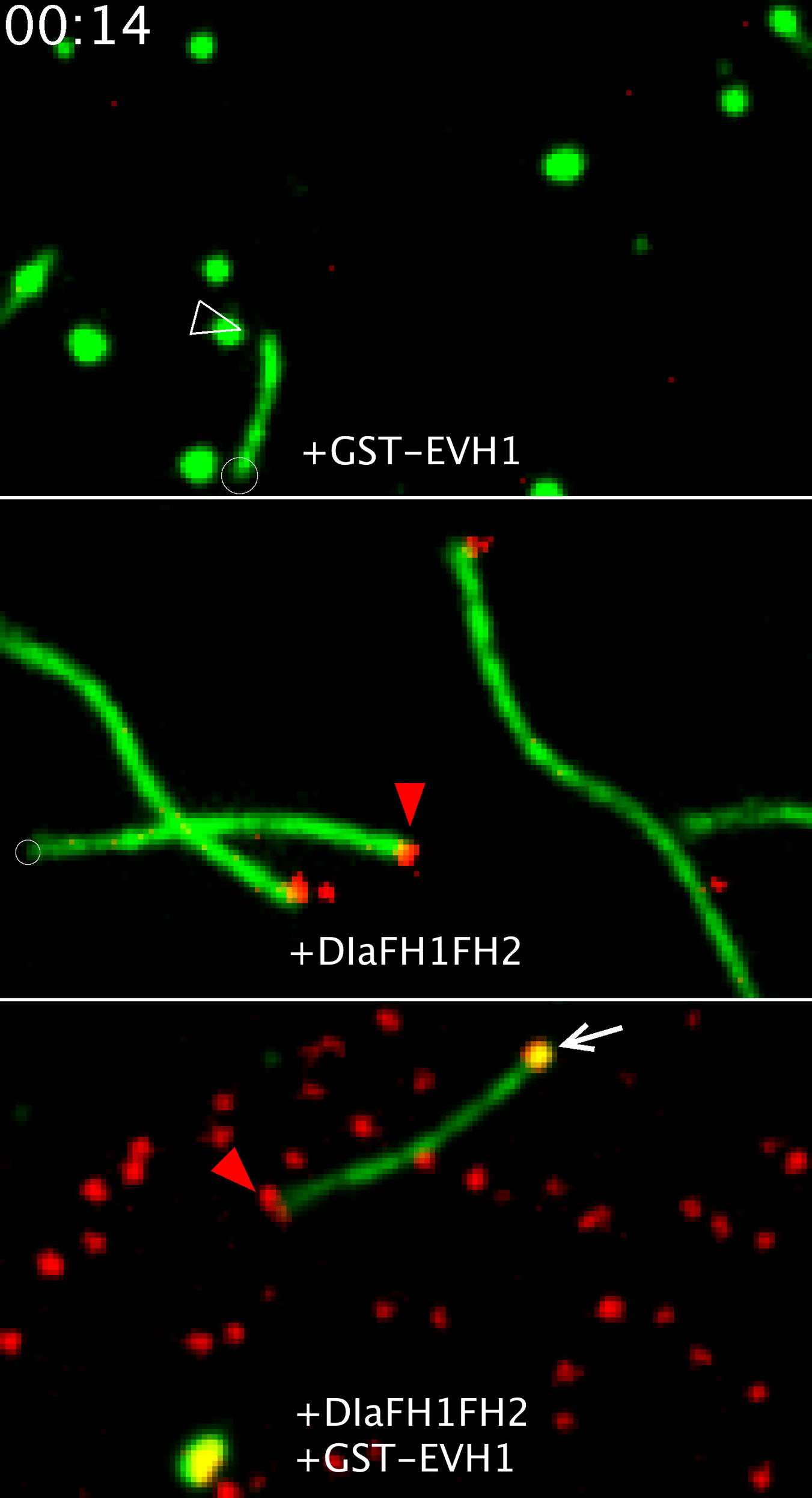

Supplement: Movie S5, Related to Figure 6. Spontaneous Actin Assembly of DiaFH1FH2, EnaEVH1, or Both in the Absence of Profilin — Top: Actin assembly is largely unaffected in the presence of EnaEVH1, although actin puncta form. TIRF movie of 1.5 μM actin (15% Oregon-green-labeled; green) + 5 μM GST-EnaEVH1 (unlabeled) in the absence of profilin. Movie shows actin puncta formation, although this has little effect on actin assembly in pyrene assays (see Figures S4A and S4B). Circle marks filament pointed end; arrowhead marks filament barbed end. Middle: DiaFH1FH2 actin assembly in the absence of profilin. TIRF movie of 1.5 μM actin (15% Oregon-green-labeled; green) + 1 nM SNAP-549-DiaFH1FH2 (red) in the absence of profilin. Circle marks filament pointed end; red arrowhead marks filament barbed end with DiaFH1FH2. Bottom: EnaEVH1 inhibits actin assembly by DiaFH1FH2. TIRF movie of 1.5 μM actin (15% Oregon-green-labeled; green) + 1 nM SNAP-549-Dia-FH1FH2 (red) + 5 μM GSTEnaEVH1 (unlabeled) in the absence of profilin. Movie shows green spots of actin accumulating in the presence of GST-EnaEVH1. Many of these colocalize with DiaFH1FH2 (red). White arrow marks EVH1/actin puncta. Eventually, a DiaFH1FH2 elongated filament emerges from the actin-DiaFH1FH2 puncta (red arrowheads). Movie is displayed at 15 frames/s. Time is min:s. [file mmc6.jpg]

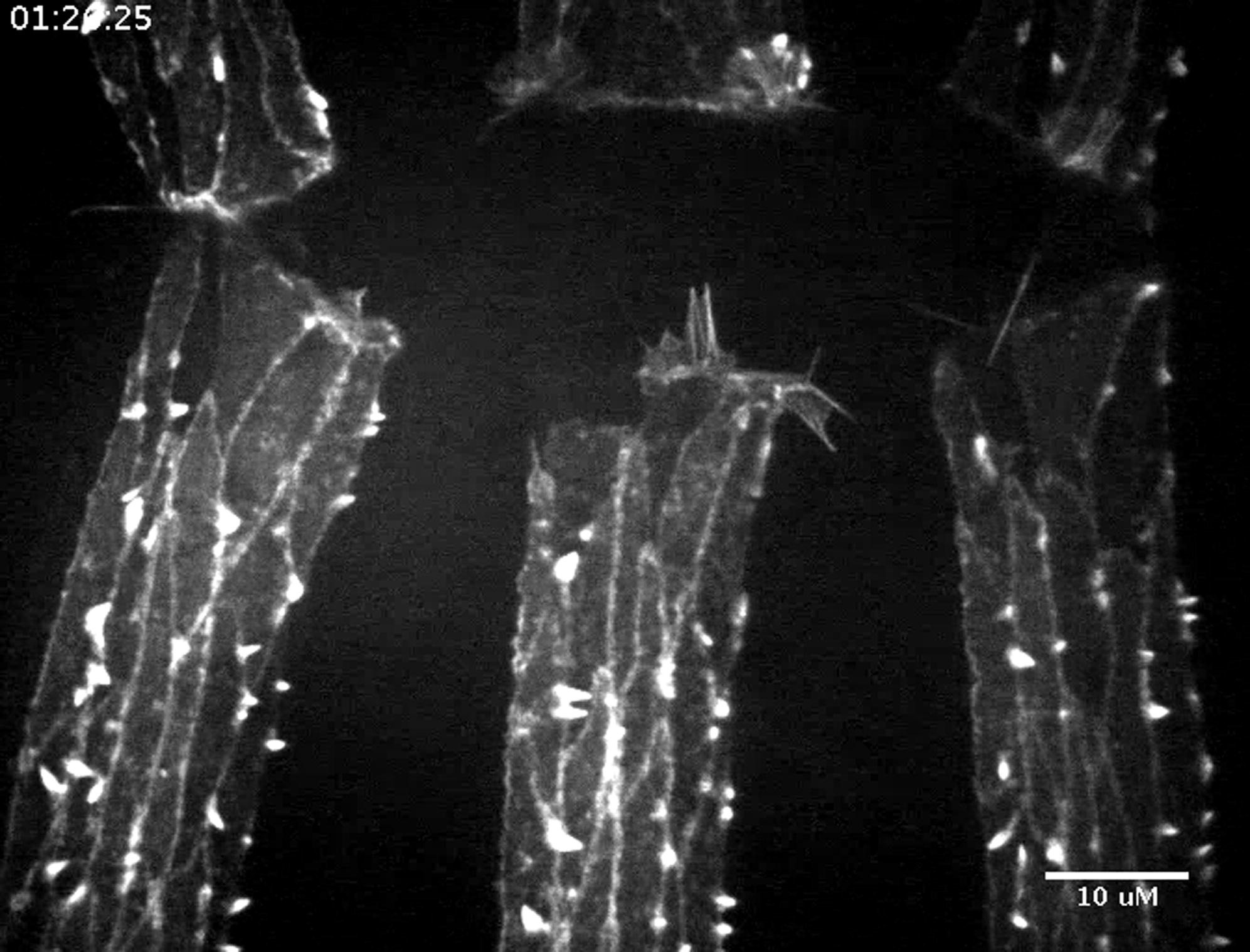

Supplement: Movie S6, Related to Figure 7. Wild-Type Drosophila Embryonic Dorsal Closure — Wild-type dorsal closure in an embryo expressing GFP-actin using engrailed-GAL4. Note endogenous Ena present. Control for Movie S7. Images (100× magnification) were collected every 5 s. Movie is displayed at 15 frames/s. Scale bar represents 10 μm. Time is hr:min:s. [file mmc7.jpg]

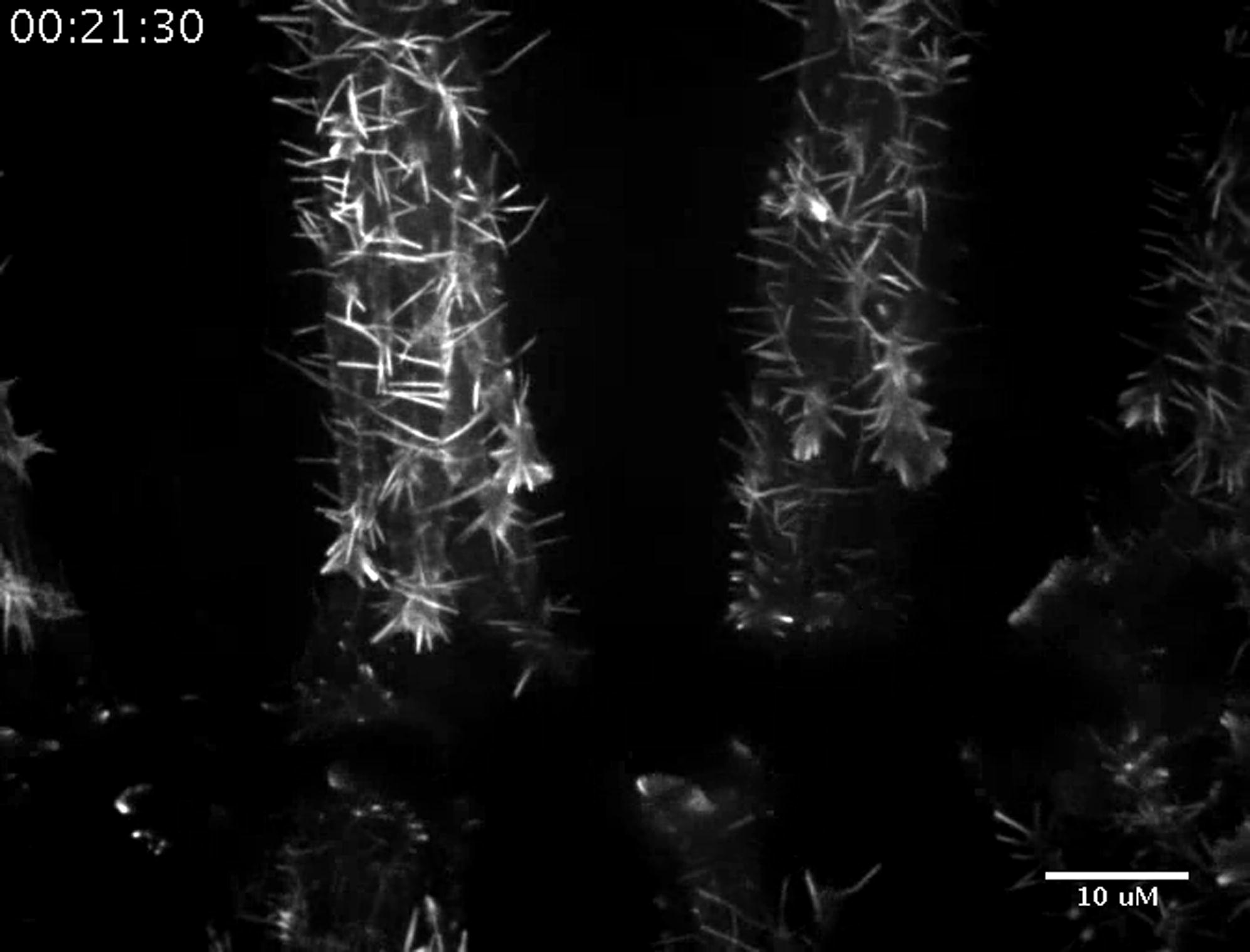

Supplement: Movie S7, Related to Figure 7. Dia-Driven Protrusions Are More Dynamic in Areas of High Ena Accumulation during Drosophila Embryonic Dorsal Closure — Dorsal closure in an embryo expressing both GFP-actin and HA-DiaΔDAD using engrailed-GAL4. Endogenous Ena is enriched at the leading edge and at tricellular junctions relative to lateral cell borders. Images (100× magnification) were collected every 5 s. Movie is displayed at 15 frames/s. Scale bar represents 10 μm. Time is hr:min:s. [file mmc8.jpg]
